# The Temporal Scaffolding of Sensory Organization

**DOI:** 10.1146/annurev-psych-032525-040352

**Published:** 2025-10-01

**Authors:** Pawan Sinha, Lukas Vogelsang, Marin Vogelsang, Albert Yonas, Sidney Diamond

**Affiliations:** 1 Department of Brain and Cognitive Sciences, Massachusetts Institute of Technology, Cambridge, Massachusetts, USA; 2 Institute of Child Development, University of Minnesota, Minneapolis, Minnesota, USA

**Keywords:** temporal regularity detection, perceptual development, simultaneity, contiguity, contingency

## Abstract

How a developing nervous system discovers meaning in complex sensory inputs has typically been examined separately for each sensory modality. Even as studies have uncovered modality-specific strategies, it remains unclear whether common principles underlie such discovery. Here, we pursue the thesis that the detection and exploitation of temporal regularities may provide a unifying mechanism for sensory organization across modalities. We synthesize research spanning neurophysiology and cognitive neuroscience and incorporate results from theoretical computer science. This integration supports the conclusion that time may be the fundamental dimension along which the brain organizes its sensorium and that the computational complexity of this problem is rendered tractable by ecologically appropriate heuristics. This proposal suggests the centrality of temporal processing in perceptual development, with implications for studies of typical and atypical development, clinical populations, and computational modeling.

## 1. INTRODUCTION

The nervous system of every infant animal is presented with, and must process, its own version of what William James famously described as “one great blooming buzzing confusion” (James 1890). An organism’s ability to successfully interact with its environment for sustenance and survival depends critically on being able to extract meaning from this confusion. Although there is a long-running debate about precisely how tumultuous a neonate’s early experience is (Piaget 1952 (1935), Slater et al. 1990, Spelke 1994, Locke 1996 (1689), Spelke & Kinzler 2007, Carey 2009, Hespos & vanMarle 2012), it does not detract from the core question of development: How does initial experience get transformed into an organized sensorium of objects and their interrelationships?

The broad objective of organizing a chaotic sensory world into a meaningful one cleaves quite naturally into three subproblems (henceforth, tasks):

1. discovering the distinct perceptual entities (or objects) that constitute the world,
2. representing these entities in such a way as to recognize them in subsequent encounters, and
3. detecting mutual dependencies between entities to be able to anticipate and plan.

Given the great variety across the animal kingdom of sensory modalities within which to accomplish these tasks (Smith 1989, Dusenbery 1992, Hughes 1999, Burnett 2011), it is plausible that the solution strategies are similarly varied to mirror this diversity. Performing these tasks in the visual domain, for instance, may rely on very different computations relative to those associated with electroception or audition. Indeed, much of the research on sensory organization thus far reflects this modality-specific approach (Bregman 1994, von der Emde 1999, Moss & Surlykke 2001, Kimchi et al. 2003, Babineau et al. 2006, Batty 2014). However, the perspective we pursue in this article is that there are likely to be unifying principles of processing that transcend the marked diversity of sensory front ends. Specifically, detecting temporal regularities in sensory streams may be a fundamental strategy by which animals, including humans, come to organize their sensorium, segmenting the world into objects, events, and relationships.

### 1.1. The Challenge of Organizing the Complex Sensorium

When an organism confronts the natural world, it encounters a barrage of sensory signals: photons hitting the retina, pressure waves vibrating the eardrum, chemical molecules detected by the olfactory system, tactile inputs on the skin, and more. Yet, notwithstanding the initial chaos, even newborn animals rapidly learn to discern meaningful units in this sensory landscape, including a parent’s face or voice, the location of a food source, or telltale signs that signal the presence of predators (Galef & Giraldeau 2001, Fischer & Frommen 2022, Mota-Rojas et al. 2022). Although some aspects of these skills may be innately available through phylogenetic dispensation, the remarkable transformation that animals undergo postnatally prompts the question: By what principles does the brain impose structure on the sensory flux, identifying recurring patterns that correspond to objects or events?

A key insight lies in the fact that stimuli are not randomly distributed in time. Instead, the environment teems with events that have regular durations, rhythms, and temporal correlations. From the repeating cycle of a parent’s voice to the predictable intervals between an approaching predator’s footsteps, temporal regularities are ubiquitous. Detecting these regularities confers clear adaptive advantages, allowing an organism to predict future occurrences, group signals that belong together, and rapidly discern relevant from irrelevant stimuli.

### 1.2. The Ubiquity of Temporal Regularity Detection Across Sensory Modalities

One reason for considering temporal regularities as a unifying principle lies in their cross-modal pervasiveness. While spatial patterns differ across sensory modalities—vision has two-dimensional spatial arrays projected onto the retina, touch arises from mechanical distortions across the skin, and chemoreception has limited spatial specificity—temporal sequencing is a universal property of neural signals. Whether an organism is dealing with a flickering light, a sequence of tones, or repeated contact with an object, the brain receives data in a temporal stream. Irrespective of its front-end provenance, the incident signal for the central nervous system is a collection of time series of neural impulses. The central nervous system faces the critical task of detecting patterns within these temporally structured inputs to generate adaptive behavior.

The external environment itself is structured in time. Many natural processes have rhythms, including diurnal cycles, seasonal shifts, and periodic motor actions (such as the beating of wings or footsteps). Socially, communication signals, such as bird songs or primate calls, also exhibit complex temporal organization, featuring repeated motifs and a hierarchical structure (Bilger et al. 2021). In addition, self-generated movements introduce predictable feedback patterns across modalities: When an animal moves, the resulting visual and auditory patterns often follow reliable temporal contingencies. Thus, temporal coherence can bind together the signals that result from a single source or event. By attending to these coherent patterns, the brain can cluster together sensory inputs that are likely generated by the same cause, laying the groundwork for perceiving objects and events. These ideas apply across all sensory modalities.

#### 1.2.1. Vision.

Although vision is often described in spatial terms, time plays a central role in many aspects of visual perception. Studies on motion detection demonstrate the brain’s ability to seamlessly integrate successive snapshots of light patterns, deriving rich visual inferences (Leslie 1984, Aslin & Shea 1990, Fiser & Aslin 2002, Sekuler et al. 2002). It is interesting to note the markedly faster developmental trajectory of temporal resolution compared with spatial resolution (Ellemberg et al. 1999a), providing temporally rich information to the infant. The phenomenon of structure from motion further demonstrates how temporally correlated points can coalesce into the perception of a coherent object even without explicit outlines or shapes. Similarly, temporal synchrony cues can bind together disparate regions of a display into one perceived object (Alais et al. 1998, Usher & Donnelly 1998, Lee & Blake 1999, Kandil & Fahle 2001). Another example derives from event segmentation, such as when perceiving distinct actions in a movie. Humans naturally parse continuous visual input at points where predictive regularities in scene dynamics shift (Baldwin & Kosie 2021), for instance, when a person transitions from walking to sitting down. These transitions are not arbitrary but typically coincide with changes in the underlying causal structure (Zacks & Swallow 2007), reinforcing the idea that the brain uses temporal cues to separate one event from another.

#### 1.2.2. Audition.

Temporal pattern detection is especially evident in how organisms carry out complex acoustic scene analysis (Bregman 1994). Speech perception in humans exemplifies this: Despite seemingly continuous air pressure fluctuations, listeners segment streams into phonemes, syllables, words, and phrases. Features such as temporal coherence, rhythmic structures, prosodic cues, and repetition are central to this segmentation (Bregman 1994, Shamma et al. 2011, de Diego-Balaguer et al. 2015). Infants, for instance, exploit statistical learning, tracking the transitional probabilities between syllables over time to discover word boundaries in continuous speech (Saffran et al. 1996, Aslin et al. 1998). Beyond human language, many animals rely on auditory temporal patterns for survival and communication. Bats use echolocation pulses and measure the time delay of returning echoes to localize prey and obstacles (Moss & Surlykke 2001). Songbirds learn and recognize songs by internalizing complex temporal structures of notes, with temporal precision and consistency exhibiting gradual developmental improvements (Glaze & Troyer 2013). These examples indicate that across taxa, detecting regularities in the timing of sounds is foundational to discerning meaning in auditory signals.

#### 1.2.3. Tactile, olfactory, and multisensory integration.

An organism moving its limbs experiences correlated changes in muscle tension, joint position, and cutaneous sensations. The temporal coupling between these signals helps to distinguish autogenous from externally generated touch. Indeed, humans exhibit sensitivity to temporal coherence in tactile stimuli, as demonstrated by the fishbone tactile illusion, wherein synchrony diminishes perceived surface indentation (Nakatani et al. 2021). Thus, temporal features can shape tactile form perception.

Even the seemingly sluggish olfactory cues exhibit marked temporal patterns. Consider a dog following a scent trail: The animal must parse the variations in concentration over time, often in conjunction with wind changes and sniffing rhythms. Similarly, the temporal sequencing of odorants, or periodicity of pulsed chemical signals (pheromones or floral scents), guides insects’ movement patterns. Insects have indeed demonstrated the ability to resolve fine-grained temporal odor patterns and utilize this information to extract spatial information (Szyszka et al. 2023). Also in humans, odor differentiation has recently been shown to occur with a temporal precision of 120 ms (Wu et al. 2024), quite in contrast to the assumed slowness of changes in this modality.

Aside from their ubiquity within each sensory modality, temporal relationships also underlie integration in a multimodal world, combining corresponding visual, auditory, tactile, and sometimes olfactory signals via dynamic correlation (Burr et al. 2009, Parise et al. 2012, Sinha et al. 2014b, Parise & Ernst 2016). This principle also underlies ventriloquism illusions, where audiovisual temporal correlation can mislead the observer about the source of a sound (Bruns 2019). Across vision, audition, touch, and other modalities, the detection of temporal regularities thus emerges as a fundamental mechanism for organizing sensory input into coherent percepts. This is not just a feature of human cognition but holds deep evolutionary significance across the animal kingdom. The classical conditioning paradigm in Pavlov’s experiments with canines, for instance, relies on the consistent temporal pairing of a neutral stimulus (bell) with a biologically relevant one (food) (Pavlov 1927). Honeybees can learn timing intervals to anticipate nectar availability in flowers (Bloch et al. 2017). Fish, amphibians, and reptiles exhibit temporal pattern recognition in contexts such as mating calls or circadian rhythms (Tosini et al. 2001, Hut et al. 2012, Pedroso et al. 2013). Mammals, including rodents, can learn to press levers at specific intervals to receive rewards, demonstrating a capacity for interval timing (Iversen 1993).

Such widespread ability hints at conserved neural substrates or computational mechanisms for temporal processing. Temporal pattern recognition underlies foraging, predator avoidance, mate selection, and social bonding. From an adaptive perspective, an organism that can predict when resources or threats are likely to appear has a survival advantage. Hence, natural selection has likely favored neural architectures optimized for extracting temporal structure from the environment.

## 2. STUDIES OF TEMPORAL REGULARITY DETECTION IN SENSORY PROCESSING: HISTORICAL FOUNDATIONS

### 2.1. Early Work in Psychophysics

Early explorations into how organisms structure the continuous flow of sensory inputs stretch back at least to nineteenth-century psychophysics. Hermann von Helmholtz (1821–1894) examined how the mind interprets sensory stimuli and argued for understanding perception as an inferential process unfolding over time (von Helmholtz 1867). By the late nineteenth century, Wilhelm Wundt (1832–1920), widely regarded as the father of experimental psychology, built upon methods pioneered by researchers such as Ernst Weber (1795–1878) and Gustav Fechner (1801–1887), establishing a laboratory dedicated explicitly to the systematic study of sensory processing and perception. Using introspection and reaction-time measurements (mental chronometry), Wundt (1913) examined the temporal characteristics of mental processes, laying the methodological groundwork for modern studies of how stimulus timing shapes perceptual experience. William James (1842–1910) emphasized the continuity of consciousness and the importance of temporal structure that situated events relative to the specious present (James 1890). He was instrumental in putting the question of sensory organization and, by extension, the role of temporal order squarely on psychology’s agenda.

### 2.2. Pavlov’s Classical Conditioning and Skinner’s Operant Conditioning Studies

An early breakthrough in connecting temporal regularity detection to learning came from Ivan Pavlov (1849–1936). His classical conditioning experiments demonstrated that dogs learned to salivate in response to a formerly neutral stimulus (a bell) when it consistently preceded the appearance of food (Pavlov 1927), showing that the temporal contiguity between stimuli drives the formation of robust associations. This underscored the importance of temporal pairing as a fundamental psychological process and set the stage for subsequent theories of timing and sequence learning in animals and humans.

While Pavlov’s classical conditioning emphasized that temporal contiguity between observed stimuli drives the formation of associations, operant conditioning, pioneered by researchers such as B. F. Skinner, highlighted that the timing between a voluntary action and its reinforcing consequence is equally critical for shaping behavior (Skinner 1938). Thus, operant conditioning involves strengthening or weakening a behavior on the basis of the consistent timing of reinforcement or punishment.

### 2.3. Gestalt Psychology and the Principle of Common Fate

Gestalt psychologists, including Max Wertheimer (1880–1943), Kurt Koffka (1886–1941), and Wolfgang Köhler (1887–1967), revolutionized the study of perception in the early twentieth century by rejecting atomistic views in favor of the idea that the brain inherently organizes sensory input into coherent wholes (Wertheimer 1923). While they primarily studied how visual principles such as similarity, proximity, and closure guide perceptual grouping, they also introduced the principle of common fate: Sensory elements changing or moving together synchronously are perceived as a unified object. This principle explicitly recognized the central role of temporal coherence in perceptual organization.

### 2.4. Lashley’s Critique on Serial Order, Hebbian Plasticity, and Spike-Timing-Dependent Plasticity

Around the mid-twentieth century, two seminal thinkers further advanced the idea that temporal structure is essential for understanding how the brain organizes behavior and perception. In his influential paper, “The Problem of Serial Order in Behavior,” Karl Lashley (1890–1958) challenged the then-prevailing reflex-chaining models, in which each action in a sequence triggers the next via feedback. He argued that such models fail to explain complex, organized sequences of behavior. Lashley proposed instead that the central nervous system anticipates and controls forthcoming sequence elements, thus inherently processing and organizing temporal structure (Lashley 1951).

Around the same period, Donald O. Hebb (1904–1985) introduced Hebbian learning, summarized by the famous maxim, “cells that fire together, wire together.” Although often interpreted in terms of spatial coactivation, Hebbian theory has key implications for timing, proposing that repeated or persistent temporally proximal activity in presynaptic and postsynaptic neurons strengthens their connection (Hebb 1949).

These ideas would later be expanded by experimental demonstrations of long-term potentiation in synaptic transmission (Bliss & Gardner-Medwin 1973, Bliss & Lømo 1973) as well as findings that both long-term potentiation and depression depend critically on the temporal order of neuronal inputs (Levy & Steward 1983). Further experimental work revealed the precise timing requirements underlying synaptic changes (Markram et al. 1997, Bi & Poo 1998) and was complemented by computational modeling (Abbott & Blum 1996, Gerstner et al. 1996). Together, this led to the establishment of spike-timing-dependent plasticity (STDP) as a fundamental mechanism, demonstrating that a presynaptic spike shortly preceding a postsynaptic spike leads to potentiation, whereas the opposite temporal order often results in depression (Song et al. 2000; for a review, see Caporale & Dan 2008). STDP thus underscores the significance of temporal contiguity, illustrating at both cellular and computational levels how the brain can encode and learn recurring temporal patterns in sensory inputs.

### 2.5. Prediction Errors, Reinforcement Learning, and Predictive Coding

An important advancement to Pavlov’s classical conditioning account came from Robert Rescorla, who demonstrated that effective conditioning depends critically on predictive relationships between stimuli, not merely temporal pairing (Rescorla 1967). He argued that the conditioned stimulus must carry informative predictive value regarding the occurrence of the unconditioned stimulus, shifting the focus from simple temporal contiguity to explicit prediction. Mathematically formalized in the Rescorla–Wagner model, learning occurs through updating on the basis of discrepancies between expected and actual outcomes, or prediction errors (Rescorla & Wagner 1972).

These insights inspired reinforcement learning theories that generalized prediction-error principles beyond classical conditioning to include learning from rewards and punishments through interactions with the environment. A reward prediction error thus guides learning by adjusting predictions on the basis of differences between anticipated and actual rewards (Sutton & Barto 1998). Temporal difference learning, introduced by Sutton (1988), thereby constitutes a specific reinforcement-learning framework where predictions are updated continuously on the basis of incremental prediction errors rather than awaiting the final outcome, rendering it particularly suitable for sequence learning.

Predictive coding emerged in the 1990s as a formal theory in which higher-level cortical regions generate expectations about incoming sensory signals, and resulting prediction errors guide perceptual inference and learning (Rao & Ballard 1999). Beyond reward-based predictions, predictive coding thus considers perception itself to be fundamentally predictive. The even-broader free energy principle (Friston 2010) later generalized predictive coding into a comprehensive framework where detecting and exploiting temporal regularities is central to how the brain minimizes surprise, thereby supporting adaptive interaction with the environment.

### 2.6. Auditory Scene Analysis and Suspicious Coincidences

Auditory perception research during the latter half of the twentieth century highlighted temporal regularity as crucial for organizing complex soundscapes. Albert Bregman pioneered auditory scene analysis during the 1970s and 1980s, describing how listeners segregate overlapping sounds into distinct auditory streams. He demonstrated that temporal coherence, such as consistent timing or rhythmic patterns, helps the auditory system determine which frequencies belong together (Bregman 1994), extending Gestalt principles into the auditory domain and highlighting the generality of temporal grouping mechanisms across sensory modalities.

A parallel and influential conceptual advancement emerged from Horace Barlow, who introduced the idea of suspicious coincidences. He suggested that sensory systems developed sensitivity to events that co-occurred more frequently than would be expected by chance (Barlow 1987, 1994). According to Barlow, detecting these coincidences constitutes a fundamental function of the brain, enabling it to identify meaningful environmental regularities while reducing redundant sensory information. This also influenced efficient unsupervised learning (Barlow 1989). Peter Földiák further extended this work from simultaneous events to sequences through his trace conditioning model, demonstrating how neurons can build invariant representations based on temporal contiguity (Földiák 1991).

### 2.7. Statistical Learning and the Infant Mind

The fundamental importance of detecting temporal regularities gained further momentum through studies of statistical learning, particularly in language acquisition research. In the late 1990s, Richard Aslin and colleagues demonstrated that 8-month-old infants are able to segment words from continuous streams of syllables by tracking transitional probabilities of how often syllables follow one another (Saffran et al. 1996, Aslin et al. 1998). Similar statistical learning abilities were subsequently shown for natural language streams (Pelucchi et al. 2009). This body of work strongly placed temporal regularity detection within developmental psychology and highlighted its role from the earliest stages of life.

### 2.8. Neurophysiology of Rhythms and Synchronization

As neuroscience methods advanced in the late twentieth and early twenty-first centuries, studies of brain oscillations and neural synchrony provided important insights into physiological mechanisms underlying temporal aspects of perception. György Buzsáki notably investigated hippocampal theta rhythms, demonstrating how periodic neuronal firing patterns could encode the timing of events (Buzsáki 2002). Wolf Singer and colleagues explored the role of neural synchrony in feature binding, proposing that synchronous neuronal firing, especially in the high-frequency bands, serves as a temporal mechanism for integrating signals belonging to the same perceptual object (Singer & Gray 1995, Singer 1999). Building on these findings, Fries (2005) introduced the communication-through-coherence hypothesis, suggesting that synchronized oscillations facilitate efficient neuronal communication across brain regions. Engel et al. (2001) similarly emphasized the functional significance of synchronized oscillations in supporting top-down predictions, proposing that they carry contextual information that shapes local sensory processing.

### 2.9. Modern Perspectives and Emerging Themes

The developments reviewed above collectively highlight how the brain relies on multiple overlapping mechanisms to detect and exploit temporal regularities. By the early 2000s, understanding temporal pattern recognition had become a focal area in perception research, prompting exploration of numerous new directions.

More recently, distinct contributions to temporal processing have been identified in specific brain regions, notably the hippocampus (Eichenbaum 2014) and cerebellum (Ivry & Spencer 2004). Moreover, the neural circuits underlying reward prediction errors, critical for learning temporal relationships, are increasingly well-characterized (Watabe-Uchida et al. 2017). Prediction errors at different hierarchical levels have also been proposed to correspond to distinct neuromodulatory systems, as revealed by hierarchical Bayesian models (Iglesias et al. 2013), advancing our understanding of perception and informing new approaches in computational psychiatry (Stephan & Mathys 2014). In addition, perception has been shown to be systematically biased toward recently experienced stimuli, demonstrating serial dependence (Fischer & Whitney 2014, Pascucci et al. 2023). Researchers have also explored how the brain manages multiple intrinsic neural timescales simultaneously (Golesorkhi et al. 2021). Finally, debates regarding discrepancies between subjective event timing and underlying neural processing delays have raised fundamental questions about whether perception reflects external “event time” or internal “brain time” (Johnston & Nishida 2001). Long-lasting temporal integration thereby provides fresh insights into the temporal structure of conscious perception (Herzog et al. 2020).

As reviewed above, the recognition of temporal regularities in sensory streams has deep historical roots, informing our understanding of phenomena ranging from auditory scene analysis to infant language acquisition. Decades of research have underscored that the brain’s remarkable capacity to structure the “blooming, buzzing confusion” of sensory inputs may fundamentally rely on detecting temporal relationships between sensory events. But precisely what kinds of temporal relationships does the brain need to detect? The structure of the real-world environment provides useful constraints for answering this question.

## 3. ECOLOGICAL CONSTRAINTS ON THE NATURE OF TEMPORAL REGULARITIES TO BE DETECTED

The natural environments in which all animals are immersed have fundamental commonalities, notwithstanding their superficial differences. The most important of these commonalities is the temporal cohesiveness of the entities that populate the environments and temporal compactness of their interactions. The first refers to the regularity that all constituents of individual objects (features, parts, views) occur in close temporal proximity to each other (Cheries et al. 2008, Rosenberg & Carey 2009, Cacchione and Amici 2015). The second refers to the temporal proximity of causes and effects during multiobject interactions (Michotte 1963, Jenkins & Ward 1965, Shanks et al. 1989, Buehner 2005). These two regularities have far-reaching consequences for the types of temporal relationships the perceptual system exploits to make sense of the world.

### 3.1. Temporal Cohesiveness of Objects: Discovering Objects Through Temporal Simultaneity and Contiguity

Since constituents of individual objects (e.g., a tiger’s stripes, sharp claws, growl, and movement patterns) typically occur in tight temporal proximity to each other, we can justifiably invert this regularity to be able to use simultaneity of features as an indication, although not a definitive one, that they may have a common provenance. Furthermore, the persistence of objects over time suggests that sensory information arriving in contiguous temporal windows may correspond to different samplings of the same underlying object. Developmental studies demonstrate that infants are able to use temporal synchrony to bind visual and auditory features into coherent objects, even when spatial cues are ambiguous (Lewkowicz 2014), and temporal proximity of occurrence to group instances (Kirkham et al. 2002).

### 3.2. Temporal Compactness of Interactions: Discovering Interobject Dependencies Through Temporal Contingencies

A second environmental regularity, temporal compactness, governs the perception of interobject relationships. Cause–effect interactions in nature, such as a ball colliding with another and setting it rolling or the rumble of sound in a subway station followed by the sight of the train, typically unfold within brief temporal windows (Jenkins & Ward 1965, Shanks et al. 1989, Buehner 2005). Psychophysical studies show that humans perceive causality most strongly when actions and outcomes are temporally proximal, even if spatial or physical contact cues are absent (Michotte 1963). Unarguably, certain relationships transpire over extended temporal intervals, such as the falling of a seed in dirt and the appearance of a shoot a few weeks later. However, most perceptual relationships that animals are sensitive to are temporally proximal to each other [Hume 1888 (1739), Shanks et al. 1989].

Given the aforementioned natural ecological constraints, we argue that the extraction of three kinds of temporal regularities in sensory data streams is required to accomplish the three critical tasks we listed in the introductory section. Simultaneity (precise temporal alignment between events) and contiguity (events transpiring in successive temporal intervals) subserve the discovery and representation of objects (Miyashita 1988; Sinha & Poggio 1996; Lee & Blake 1999; Wallis & Bülthoff 2001; Sinha et al. 2009, 2014b; Jesse & Johnson 2016), while contingency (temporal noncontiguity, but probabilistic consistency between event occurrences) enables the detection of mutual dependencies between objects (Watson 1966, 1984; Sternberg & McClelland 2012; Pezze et al. 2016; Jacquey et al. 2020). This mapping between temporal regularities in the environment and tasks to be performed highlights the importance of understanding the temporal structure of incoming sensory data. Underscoring the significance of these temporal regularities, a growing body of empirical evidence that we review below reveals the encoding of these relationships in multiple neural sites in the mammalian brain.

### 3.3. Neural Correlates of Simultaneity Detection

Detecting whether sensory events occur simultaneously or sequentially involves multiple processes spanning both subcortical and cortical structures. In typical experimental paradigms probing these processes, two stimuli (within or across sensory modalities) are presented with varying temporal delays, and participants are asked either to judge whether the stimuli appeared at the same moment (simultaneity judgment) or to indicate which stimulus came first (temporal order judgment). Corresponding neuroimaging studies, frequently conducted within multisensory contexts, have identified several brain regions that contribute to these judgments in complementary ways.

Subcortically, the superior colliculus contains multisensory neurons that respond specifically when inputs from different modalities coincide in time (Meredith & Stein 1986). These cells can exhibit enhanced (or occasionally suppressed) firing when stimuli are presented together versus separately. As such, they provide a neural signature of temporal coincidence detection and may guide rapid orienting responses toward synchronous sensory events. Cortically, a widely distributed network appears to mediate the detection of stimulus synchrony. For example, audiovisual asynchrony detection activates the insula, posterior parietal, prefrontal, and cerebellar areas in humans (Bushara et al. 2001), with the insula showing particularly pronounced activation, suggesting that it helps process temporal cues at an early stage within the cortical processing hierarchy. Further, in tactile simultaneity judgment, the striatum plays a role in estimating intervals between stimuli, while the right inferior parietal lobule appears to specifically discriminate simultaneous from nonsimultaneous stimuli (Kimura et al. 2019).

Dissecting the various components underlying temporal judgments, functional magnetic resonance imaging recordings during an audiovisual correspondence detection task reveal modulation of inferior frontal and superior temporal cortical areas by temporal task complexity. In contrast, the dorsolateral prefrontal cortex (DLPFC) is modulated by stimulus asynchronies impacting memory load (Baumann et al. 2018). Moreover, despite both tasks involving assessments of temporal relationships, simultaneity and temporal order judgments appear to recruit partly distinct neural mechanisms, with temporal order judgments requiring an additional processing stage to determine the sequence of events. Specifically, neuroimaging studies on audiovisual timing tasks have shown that prefrontal, parietal, and occipitotemporal regions of the left hemisphere are differentially engaged during temporal order judgments, reflecting these added task demands (Binder 2015, Love et al. 2018).

### 3.4. Neural Correlates of Contiguity Detection

Contiguity refers to stimuli occurring not exactly at the same time, but in close temporal proximity. As described above, classical conditioning experiments have demonstrated that repeated temporal pairing of a neutral stimulus (e.g., a tone) with an unconditioned stimulus (e.g., a puff of air to the eye) can lead to learned associations (Pavlov 1927, Rescorla & Wagner 1972). Interestingly, strict simultaneity (presenting both stimuli at precisely the same time) typically results in markedly weaker conditioning than having the neutral stimulus precede the unconditioned one (Spooner & Kellogg 1947, Fitzwater & Reisman 1952), highlighting the importance of temporal contiguity perception.

At the synaptic level, STDP, as introduced in Section 2, is able to account for this phenomenon by demonstrating that when a presynaptic spike precedes a postsynaptic spike within a short temporal window, long-term potentiation occurs, with a slight lag between the two being more effective than precise simultaneity at inducing potentiation, while reversed timing can drive depression (Markram et al. 1997, Bi & Poo 1998, Dan & Poo 2004, Caporale & Dan 2008). Thus, STDP offers a fine-grained neural mechanism by which firing patterns minimally separated in time, but not strictly coincident, can become associated.

Several brain regions support the detection and processing of temporally contiguous sensory information. Neurons in the anterior ventral portion of monkey inferior temporal cortex are sensitive to stimuli presented in close succession, with some cells maintaining activity over time, thereby able to link events within short-term memory (Miyashita 1988). The hippocampus exhibits a remarkable facility to encode temporal sequences. In part, this appears to be accomplished through specialized time cells (MacDonald et al. 2011, Eichenbaum 2014) that fire in relation to specific moments and thus help link successive events as well as more temporally separated ones (also relevant for contingency detection, as covered further below). Although classical conditioning itself does not depend on the hippocampus, this structure is necessary for trace classical conditioning, where a temporal gap separates the conditioned and unconditioned stimuli (Solomon et al. 1986, McEchron et al. 1998). This requirement has been explicitly attributed to the absence of direct temporal contiguity in trace conditioning (Bangasser et al. 2006), indicating the important role of the hippocampus in bridging stimulus information across temporal delays. Moreover, the cerebellum contributes to precise representations of short temporal intervals between successive events, aligning with evidence that it supports fine temporal precision, notably in motor-related contexts (Ivry & Spencer 2004). Finally, consistent with its role in implicit associative learning, the striatum (particularly the right caudate) is activated during statistical learning of structured sequences (Turk-Browne et al. 2009), which, depending on the time scale, may also relate to contingency detection.

### 3.5. Neural Correlates of Contingency Detection

Detecting long-range probabilistic dependencies between sensory events is more complex than detecting simultaneous events or learning short-interval associations. This process involves, among others, memory systems capable of bridging extended temporal gaps and prediction and learning circuits that dynamically track long-term statistical regularities.

As introduced above, the hippocampus prominently encodes and retrieves temporal sequences and contains specialized time cells capable of bridging intervals separating events (MacDonald et al. 2011, Eichenbaum 2014). Through such consequent relational memories, the hippocampus can link contexts or stimuli encountered at different times. Additionally, the DLPFC is able to maintain relevant information in working memory (Curtis & D’Esposito 2003), thereby enabling direct comparisons between earlier and later events. Together, these memory capacities are important for identifying delayed or probabilistic cause-and-effect relationships.

With regard to prediction, contingency learning is dependent on neural circuits that compute prediction errors. Midbrain dopaminergic neurons signal these prediction errors by responding to unexpected rewards, shifting their activity from rewards themselves to predictive cues, consistent with reinforcement learning theories (reviewed in Schultz 1998). Human neuroimaging studies also demonstrate that the ventral striatum and associated areas encode prediction errors in line with temporal-difference learning models (O’Doherty et al. 2004). Overall, these circuits, together with the involvement of the hippocampus providing a possibly long-term temporal bridge via memory, the DLPFC maintaining relevant information in working memory, and additional brain regions, support the detection and learning of long-range probabilistic dependencies within the sensory environment.

Against the backdrop of this empirical evidence of nervous systems’ sensitivity to simultaneity, contiguity, and contingency relationships, we next provide brief formal specifications of the tasks to be performed and characterizations of their complexity. This treatment corresponds to the first two of Marr’s levels of analyses. We then consider the likely heuristics the nervous system might adopt in order to overcome the computational challenges of the task demands.

## 4. FORMAL TASK STATEMENTS AND COMPLEXITY ANALYSES

### 4.1. Simultaneity Detection: Spotting Co-Occurring Object Features

Imagine sampling the world with several feature detectors at multiple time instants. All sensed features in any one sample are, by construction, simultaneous. An object exists in a subset of the samples. From the full collection of samples, the challenge is to discover this object. We can formalize this problem definition as follows.

#### 4.1.1. Problem statement.

**Figure 1:**
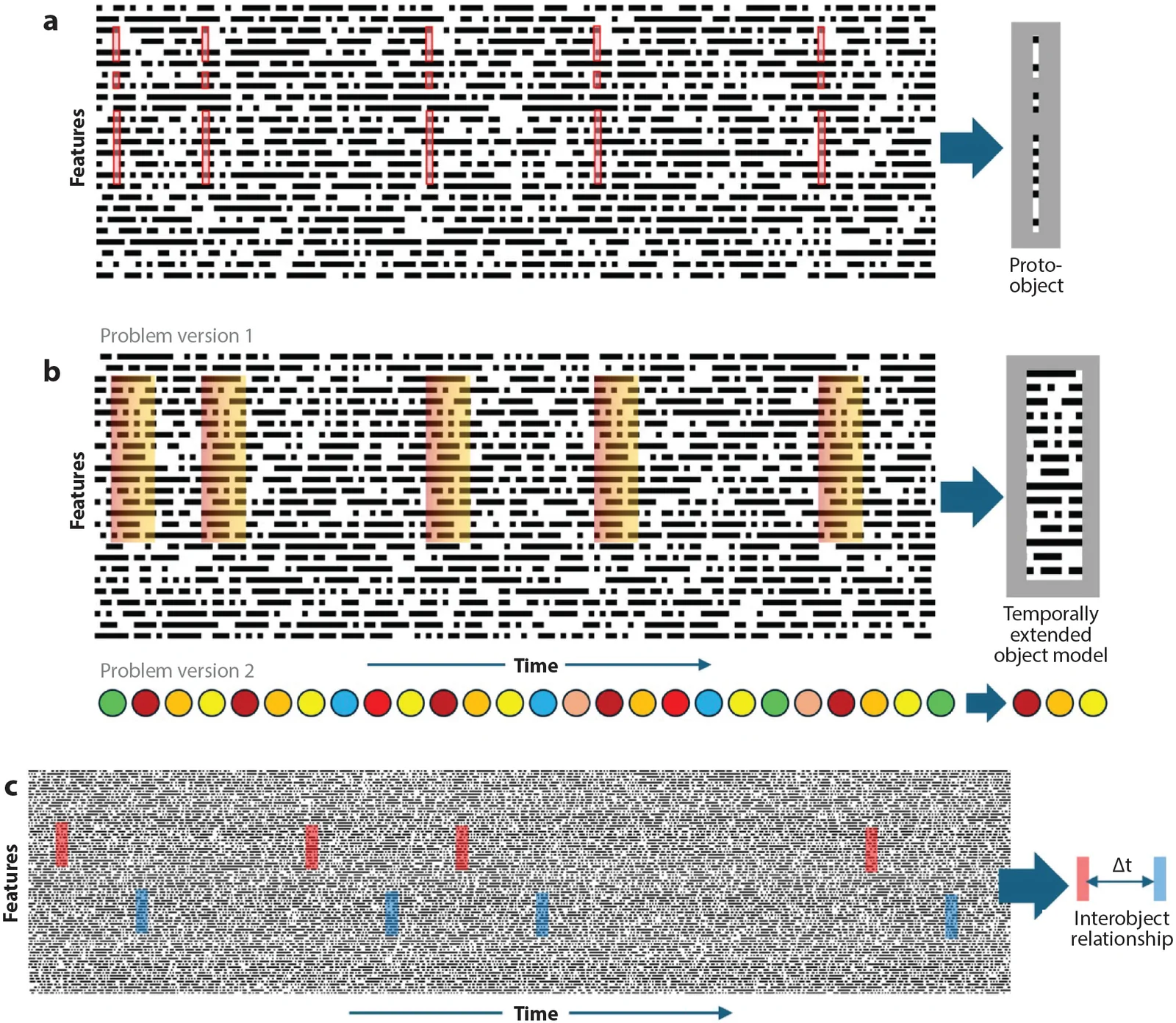
Schematic depiction of the basic tasks associated with the three kinds of temporal relationships. (*a*) The problem of simultaneity detection, framed as one of repeated substring discovery. (*b*) Two versions of the contiguity detection task, both of which require the binding of multiple sensory patterns, via temporal proximity cues. (*c*) Contingency detection involves detection of consistent probabilistic dependencies between discovered objects, even though the objects may be separated by significant temporal durations.

Assume a world with discrete time instants. (This is an assumption that will be shared across all three problem domains, e.g., simultaneity, contiguity, and contingency detection. It simplifies problem exposition and complexity analysis without loss of generality.) In this world, assume the availability of *m* feature detectors that signal in a binary fashion the presence or absence of their preferred feature. The state of the sensed world at any one time instant then is defined by an *m*-long binary vector, with its on-bits indicating which features were present at that instant. The longitudinal status of the world across *n* time instants is then expressible as an *m × n* binary matrix. In this world, an infrequently present object corresponds to a binary string of length *k* (*k* < *m*) that is present in some subset *r* of the *n* world states. The elements of this *k*-long substring occur simultaneously with each other. But there are also spurious simultaneous co-occurrences of features unrelated to the object. The task for a learner is to discover what the repeating substring is from the *m × n* matrix, even as the length of the substring, its location within the full *m*-long string, and its number of repeats are all unknown a priori. Figure 1a illustrates this formal version of the problem.

#### 4.1.2. Solution strategies.

As formulated, this problem maps closely onto the classic longest common subsequence (LCS) problem (Paterson & Dančik 1994). The simplest case of finding the LCS across two strings is tackled via dynamic programming (DP). If the two strings have length *p* and *q*, the DP approach can yield a polynomial time complexity of *O*(*pq*). However, for larger numbers of strings, the complexity increases rapidly and the problem becomes NP-hard, with no known polynomial-time solutions. In fact, the real challenge is even worse. The multistring formulation of the LCS problem assumes that the subsequence is common to all of the input strings. Since, in our case, only a subset of the strings is expected to have the shared subsequence, we do not even know which subsets to consider. This version of the problem is referred to as a partial-coverage LCS or a threshold LCS, where the threshold refers to the proportion of the input strings that are guaranteed to have the subsequence and is, in general, more complex than full-coverage settings.

The reason for partial-coverage LCS’s greater complexity is that there are 2^*n*^ potential subsets of *n* strings. A naive approach might try all subsets of strings, solve an LCS for each subset, and pick the longest. This quickly becomes intractable as *n* grows because of the exponential blow-up in the number of subsets to consider. No good solution strategies exist. In light of the computational intractability of the partial-coverage LCS task, it is notable that the brain is able to solve the problem adequately in the real world. After presenting formal statements of the other two problems, we describe in Section 5 potential heuristics that the nervous system might adopt in order to render a solution to the problem feasible.

### 4.2. Contiguity Detection

Objects typically have some temporal persistence. Instead of transiently blinking in and out of existence, they reveal different aspects of their appearance (say, different views of a 3D object) over short spans of time. These aspects are candidates for linkage given their mutual temporal proximity.

#### 4.2.1. Problem statement (version 1).

Assume the same problem statement as for the simultaneity detection case, that is, an *m × n* binary matrix that represents observed features over *n* time steps. In this matrix, after having determined the repeated substring corresponding to an object, we need to determine the temporally adjacent substrings that correspond to slightly transformed instances of the object.

#### 4.2.2. Problem statement (version 2).

If we assume that the simultaneity-based object-discovery step has already been accomplished, and a *k*-long subsequence has been identified, let us consider an alphabet defined over *k*-long binary sequences. There are 2^*k*^ letters in this alphabet. Now imagine an *n*-long sequence drawn from this alphabet. The task of contiguity detection is to determine the letters that are temporal associates of the discovered object.

Both versions are illustrated in Figure 1b. For ease of exposition, and since we have separately considered the problem of simultaneity-based object discovery, we work with version 2 of the problem.

#### 4.2.3. Solution strategies.

As formulated, this problem maps closely to bigram learning in sequences. The solution is straightforward, requiring just one pass over the sequence. In a sequence of length *n*, there are *n* − *1* consecutive pairs (bigrams); one can process each pair exactly once in a single pass, updating counts in a dictionary (hash map) or another suitable data structure for each bigram encountered. In a hash map, each insertion/update is *O*(1) on average. Therefore, the total time is *O*(*n*) × *O*(1) = *O*(*n*). In terms of memory usage, the maximum number of distinct bigrams is bounded by the square of the vocabulary size (i.e., *V*^2^ if *V* is the vocabulary size). Storing counts for each distinct bigram typically requires *O*(*V*^2^) in the worst case (if every possible pair occurs). It is worth noting that since the approach requires only a forward pass, it is adaptable to a streaming scenario, maintaining bigram counts in an online fashion, adding one bigram at a time. The amortized complexity remains *O*(*n*) for a sequence of length *n*.

### 4.3. Contingency Detection

Distinct objects, within or across modalities, may exhibit probabilistic relationships with one entity that appears following some temporal lag after the other with greater consistency than would be expected by chance. Unlike in the case of contiguity relationships, here the lags can be arbitrarily long. Perhaps one of the most notable examples of contingency relationships is the association between Pavlov’s bell and the subsequent appearance of food for his canine subjects. Detecting such relationships confers great adaptive benefits, since it enables the creation of a world model that can underlie prediction.

#### 4.3.1. Problem statement.

Imagine an *n*-long sequence where each element is drawn from an alphabet with cardinality *m* (and *n >> m*, i.e., the number of observations is much greater than the size of the alphabet). The problem is to detect probabilistic dependencies between any two elements (with *k* as the upper bound on how far apart they can occur in the input sequence) based on observing the sequence.

#### 4.3.2. Solution strategies.

The goal is to estimate how often a symbol at some position co-occurs with another symbol at some later position when the difference in positions can be as large as *k*. In principle, this can be achieved through an elaboration of the strategy proposed for the task of contiguity learning (where *k* is constrained to be 1). Imagine constructing a 3D matrix of size *k × m × m* and initializing all of its entries to zero. As each element of the *n*-long sequence is encountered, all *k* of its antecedents are examined. If the current element has index *i* in the alphabet (1 ≤ *i* ≤ *m*), and an antecedent *r* steps away has alphabet index *j*, then element <*r*, *i*, *j*> is incremented by 1. After considering the entirety of the input sequence, matrix entries that have values significantly higher than what would be expected by chance for a random sequence indicate the elements that are likely to be probabilistically linked as well as the temporal gap between the two. Computation of the time complexity of this process is straightforward. For each element of the *n*-long input sequence, we perform *k* operations, yielding a complexity of *O*(*nk*). The space complexity is proportional to the size of the 3D matrix that needs to be maintained to keep track of interelement relationships. For a [*k × m × m*] matrix, this is *O*(*km*^2^).

## 5. POTENTIAL HEURISTICS TO REDUCE COMPUTATIONAL COMPLEXITY AND THEIR USAGE BY THE BRAIN

The problem and algorithmic descriptions above provide a broadly instructive indication of the computational and memory demands associated with detecting the three kinds of temporal regularities in the general case. We next consider possible heuristics that can reduce this complexity. It is interesting to note that these heuristics predict strategies that can be, or are known to be, deployed by biological systems. The computational analysis of these tasks, thus, provides a principled way to explore potential solutions and simplifications as implemented in biology.

### 5.1. The Simultaneity Detection Task

Object discovery, which involves the detection of repeated subsequences (whose components are linked via simultaneity) across multiple time instances, is a challenging NP-hard task in the general case. What kinds of heuristics can make this problem tractable? Below, we describe some possible simplifications. Interestingly, each of these heuristics can be motivated by ecological constraints and/or maps onto strategies that are believed to be used by biological perceptual systems.

#### 5.1.1. Enforcing locality of features.

Instead of allowing the subsequences to be noncontinuous, we can constrain them to be unbroken. This reduces the task to a longest common substring problem rather than the more complex LCS problem (Gusfield 1999). The notion of locality of features can be justified by the spatial cohesiveness of objects in our natural environment. All of their constituent elements occur in close and continuous spatial proximity. A corresponding bias in biological systems would predict that we would be best able to detect simultaneity relationships between spatially proximal sensory elements without intervening extraneous features. While direct empirical validation of this prediction is currently lacking, it appears to be a straightforward question to operationalize as an experiment.

#### 5.1.2. Reducing cardinality of features.

The fewer the features over which to search for a common substring, the lower the computational complexity would be. This idea of reducing cardinality of features may relate quite directly to the known developmental progressions in biological systems. For instance, human infants are born with strikingly poor visual acuity (Dobson & Teller 1978). This lack of high-resolution information in the early visual signals from the eye to the brain may significantly reduce the number of distinct features over which to run the object discovery computation. In separate lines of investigation, we have explored how this initial degradation in acuity, and various other perceptual dimensions such as color, may play adaptive roles for instantiating robust representations for encoding natural visual information (L. Vogelsang et al. 2018, 2024; M. Vogelsang et al. 2024). We hypothesize that a similar process may operate in prenatal hearing (Vogelsang et al. 2023).

#### 5.1.3. Identifying relevant features.

The identification of relevant features within the large constellation of all sensed features is perhaps the most significant simplification of the task. Certain assumptions about which features are likely to be part of the eventual object representation would reduce the challenge of object discovery via an accumulation of co-occurrence statistics. This possibility, in effect, argues for a way to solve the problem of object discovery in one shot. The great data hunger of current artificial intelligence (AI) systems, such as convolutional neural networks, tasked with object discovery (e.g., Alzubaidi et al. 2021) points to their not using such a simplifying heuristic. Humans, on the other hand, often show far more rapid object learning than AI models, sometimes reducing to one-shot learning (Lake et al. 2011). What strategies might underlie such profound efficiency on the object discovery task? A potential answer lies in the dynamics of the inputs. As a consequence of structural cohesion of real-world objects, all elements of an object share the same overall movement trajectory. Conversely, the lack of physical connectedness of distinct objects typically ensures that the movement trajectories of the elements of one object will not consistently be shared by those of another. Hence, similarity of movement vectors across features provides a powerful cue to bind subsets into potential object assemblies. This observation is reminiscent of the Gestalt proposal of using common fate as a grouping mechanism (Wertheimer 1923). The usage of dynamic cues provides for one-shot object discovery and largely dispenses with the need to have to memorize numerous static instances over which to detect co-occurrence statistics and thus discover objects. Given the great computational simplification motion yields, it is perhaps not surprising that every animal exhibits sensitivity to dynamic sensory cues. Developmentally, motion cues are the first to signal object unity, well before any of the figural cues, such as similarity of color, texture, or contour alignment, are effective (Kellman & Spelke 1983).

To summarize, the brain might be able to overcome the significant computational complexity of the simultaneity-driven object discovery task by resorting to heuristics that lead to simplification of the input sensorium, privilege spatially proximal relationships, and use object dynamics to enable one-shot learning and reduce, or even eliminate, the need for extensive co-occurrence statistics accumulation.

### 5.2. The Contiguity Detection Task

While the computational and space complexity of the solution strategy for contiguity detection appears quite modest [*O*(*n*)], it is important to note a few implicit assumptions. First, the approach assumes a high-fidelity storage that can keep track of all bigrams encountered, whether spurious or genuine. For even modest values of alphabet cardinality, this can prove to be a heavy demand. Second, the approach arbitrarily restricts contiguity detection to bigrams and does not cover trigrams or more. How might one use transitivity to create more comprehensive equivalence classes, but not so large as to incorporate extraneous associations? What heuristics might the brain adopt to address these issues?

A powerful heuristic involves incorporating similarity of instances in the association process. This idea builds on a prominent result from the domain of classical conditioning, that related stimuli are more readily associated than unrelated ones (Rescorla 2008). In the context of sensory organization, this translates to deploying temporal contiguity-based association to perceptually similar stimuli. In terms of the solution strategy described above, this is akin to reducing the *m × m* matrix to one that is a diagonal band (assuming that the elements are ordered along the rows and columns of this matrix according to their mutual similarities). This significantly reduces the space complexity of the solution since only a small subset of the matrix now needs to be stored. The proposal also addresses the issue of runaway association by indiscriminately linking all contiguous elements. Since only similar perceptual instances are to be grouped by this heuristic, a drop in similarity between the instance at one time point and the next one terminates the association process. Although we currently lack systematic experimental evidence regarding how association strength changes as a function of perceptual similarity of temporally contiguous elements, several studies of temporal association implicitly demonstrate the need for such similarity (Sinha & Poggio 1996, Harman & Humphrey 1999, Wallis & Bülthoff 2001, Cox et al. 2005, Liu 2007, Wallis et al. 2009).

In summary, the temporal contiguity-based association task, though straightforward in terms of time complexity, poses a challenge with respect to storage complexity. However, using instance similarity as a heuristic for conditioning association can help with the memory resource demands and also provide a natural strategy for limiting the transitivity-induced extent of grouping. Evidence from a few past studies and the broader domain of classical conditioning provide justification for this heuristic.

### 5.3. The Contingency Detection Task

As is the case with the contiguity detection task, contingency detection involves keeping track of co-occurrences of objects across different temporal gaps. As the allowable temporal gap increases in duration, memory demands grow since many more objects can make an appearance during extended periods and become candidates for linkage. The size of the 3D matrix, which is *km*^2^, with *k* as the maximum temporal separation between two objects and *m* as the number of objects in the database, increases as *k*, and hence *m*, increases. Given the brain’s limited working memory capacity, maintaining the full matrix veridically is impractical. A few heuristics may serve to reduce memory demands of the contingency detection task. The basic motivation for the heuristics is evidence demonstrating that working memory likely maintains a trace of only a few of the most salient objects encountered during immediate, recent, and long past periods (Cowan 2010), and the veridicality of an object’s timestamp gets progressively coarser the further in the past the object has appeared (Mau et al. 2018, Taxidis et al. 2020). With these constraints, the size of the putative matrix that needs to be maintained is greatly reduced, via a reduction in both the object set cardinality (*m*) and the number of time lags that need to be represented (*k*). Two predictions this heuristic makes are as follows: (*a*) probabilistic dependencies will be detectable only between salient objects, and (*b*) for salient objects separated by long lags, the detected relationship will likely not encode precise lag values. Studies are needed to empirically test these predictions. It is also worth noting that for humans, symbolic representations such as language may vault objects seen long ago into visual awareness (Lupyan & Ward 2013). Thus, they may act as temporal wormholes, enabling a seeming shortening of the actual temporal distance between them and more recently viewed objects, thereby facilitating the detection of contingency relations between them.

## 6. BROADER ISSUES

### 6.1. How Capable Are Current Computational Models of Perception and Cognition, Specifically Deep Networks, at Detecting Different Temporal Regularities?

The key tasks described in this review are centered around discovering objects in the environment, refining their representations across different instances, and understanding their interrelationships. How well do current AI models perform on these tasks? In the visual domain, object recognition has typically relied on deep convolutional neural networks (CNNs) such as the AlexNet (Krizhevsky et al. 2017) and many elaborations, trained on large, labeled image datasets. These CNNs excel at extracting static spatial features, such as edges and textures, to classify objects in still images. However, by design, they lack an inherent time axis and, thus, fail to capture dynamic changes over time, such as motion, evolving viewpoints, or sequential interactions.

To overcome some of these limitations and allow networks to process video sequences rather than only static images, conventional CNNs have been extended into the temporal domain. Such 3D-CNNS convolve over both spatial and temporal dimensions, enabling them to capture temporal cues, such as simultaneity and contiguity, across successive frames. However, as 3D-CNNs typically operate over fixed and short temporal windows, they are limited in their ability to model long-range dependencies. Recurrent neural networks (RNNs), and especially long short-term memory (LSTM) networks, which can maintain internal states over longer sequences, offer more temporally extended processing. This capability may be even more pronounced in transformer-based architectures that, via self-attention mechanisms, enable direct connections between distant events in a sequence, making them potentially most suitable for modeling contingency (for comparisons between different models, see Manttari et al. 2020, Alomar et al. 2024).

While many spatiotemporal architectures were initially developed for supervised tasks such as action recognition, recent work in unsupervised and self-supervised learning has begun to explore object discovery without heavy reliance on labeled data. For instance, inspired by human development, Orhan & Lake (2024) demonstrated that self-supervised neural networks trained on naturalistic egocentric video data from the viewpoint of a child can learn high-level visual representations that capture broad semantic categories. Similarly, based on audiovisual recordings from a child’s perspective, Vong et al. (2024) showed that multimodal learning frameworks, which align visual and linguistic representations based on temporal co-occurrence, can successfully acquire word–referent mappings. This suggests that key aspects of early word learning can be achieved through domain-general simultaneity-based associative learning.

These approaches illustrate the potential of using temporal information for object discovery and also underscore how insights from human development can enhance machine learning systems (Zaadnoordijk et al. 2022). They highlight the need to consider temporal dynamics even in tasks such as object recognition that have traditionally been viewed as relying primarily on the static spatial structure of images.

### 6.2. Can Some Clinical Conditions Be Understood as Compromises in Temporal Regularity Detection? If So, What Are the Implications for Their Diagnostics and Interventions?

If temporal regularity detection indeed constitutes a foundational building block for organizing the sensorium, its disruptions may significantly impact perception and cognition across various clinically significant conditions. This association between atypicalities in temporal processing and neurological, neuropsychiatric, and/or developmental conditions appears to find support in clinical observations.

In the case of schizophrenia, impaired temporal processing can disrupt the sequencing of perceptual events, leading to fragmented subjective experience and a compromised coherence of the sense of self (reviewed in Giersch 2024). This positions timing dysfunction at the phenomenological core of schizophrenia. Empirical evidence indicates that patients face challenges in estimating temporal durations (reviewed in Ciullo et al. 2016) and require larger temporal gaps between sensory events to perceive them as sequential rather than simultaneous (Foucher et al. 2007; Giersch et al. 2009, 2015). This significantly affects the patients’ comprehension of sensory event structures. Beyond short-scale simultaneity perception, deficits also occur in perceiving temporal contiguity between actions and their consequences, disrupting the patients’ sense of agency (Roth et al. 2023). In light of growing evidence linking timing impairments to perceptual and cognitive dysfunction, targeted sensory training and technology-based intervention may offer promising avenues for improving clinical diagnosis and treatment (Amadeo et al. 2022).

Individuals with autism, in contrast, exhibit intact or even enhanced temporal resolution, characterized by narrower simultaneity windows in the visual domain observed in both adolescents/adults (Falter et al. 2012) and toddlers (Freschl et al. 2021). Such heightened temporal resolution may enable faster parsing of temporal events and support a more detail-focused perceptual processing style. However, when sensory integration spans multiple modalities, broader temporal integration windows have been reported (reviewed in Zhou et al. 2018), suggesting compromised cross-modal sensory integration. Autistic individuals have also been shown to overestimate the volatility of the sensory environment (Lawson et al. 2017) and have been associated with difficulties in higher-order predictive processing across longer temporal intervals (Sinha et al. 2014a, Cannon et al. 2021). This raises the possibility that therapies emphasizing temporal training, such as music therapy or rhythmic entrainment, could potentially enhance sensory integration and temporal prediction.

Individuals with other clinically relevant conditions also exhibit distinctive deviations in temporal processing. For instance, attention-deficit/hyperactivity disorder (ADHD) involves impaired time estimation and discrimination, frequently accompanied by the sense of having insufficient time to complete tasks, thereby directly impacting executive functioning (Ptacek et al. 2019). Notably, pharmacological treatments improve both ADHD symptoms and temporal processing, suggesting that timing deficits could serve as potential diagnostic markers and treatment targets (Ptacek et al. 2019). Similarly, Parkinson’s disease is characterized by deficits in temporal processing, manifested through elevated simultaneity thresholds and impaired multisensory integration (Artieda et al. 1992, Rostami et al. 2024). These timing deficits have been linked to disruptions in dopaminergic signaling and processing in frontal areas, emphasizing the potential relationship between temporal processing and executive dysfunction (Parker et al. 2013).

Localized cortical damage can lead to very evident loss of sensory or motor functions. However, it can also result in less immediately apparent challenges to temporal processing. For instance, patients with Balint’s syndrome experience simultanagnosia, characterized by the inability to perceive multiple stimuli presented simultaneously (Kinsbourne & Warrington 1962). Such patients show improved perception with longer stimulus exposure. Additional phenomena reflecting temporally disrupted perceptual processing include palinopsia and palinacousis, visual and auditory disturbances characterized by pathological temporal persistence or recurrence of sensory experiences (Critchley 1951, Diamond & Bender 1965, Bender et al. 1968, Jacobs et al. 1973). Unlike ordinary afterimages, these sensory phenomena often persist independently of eye movements, differ chromatically and spatially from the original stimuli, and may correspond electrophysiologically to seizure-like activity. Another relevant example is sensory extinction, where simultaneous bilateral stimulation leads to reduced or lost perception of the stimulus contralateral to unilateral cerebral lesions (Bender & Furlow 1945, Bender & Teuber 1946, Bender 1952). Crucially, extinction illustrates how perceptual interactions are influenced by stimulus intensity and temporal order: Presenting stimuli first to the impaired side preserves perception, while reversing this temporal order significantly worsens perceptual deficits.

Collectively, these selected examples illustrate how disruptions in temporal processing can manifest across diverse deviations from typical sensory functioning, underscoring the clinical potential of temporal assessments for diagnostic and therapeutic interventions.

### 6.3. Are There Ecologically Relevant Regularities That Lie Beyond the Temporally Focused Taxonomy? If So, How Might the Nervous System Detect Them?

Although we highlight the importance of temporal regularity detection, it is important to acknowledge that beyond temporally centered learning, the nervous system also supports the acquisition of knowledge based on spatial, relational, and abstract regularities. A comprehensive view of learning must therefore encompass both temporal and non-temporal factors. Several prominent learning phenomena do not necessarily depend on the detection of temporal regularities.

Insight learning, for instance, involves sudden problem solving through cognitive reorganization, as famously demonstrated by Köhler’s experiments in which chimpanzees, such as Sultan, unexpectedly used tools in novel ways to obtain food, suggesting a cognitive “eureka” or “aha” moment rather than a gradual accumulation of knowledge (Köhler 1925). Although the precise mechanisms remain to be fully elucidated, emerging studies have begun to implicate processes such as coarse semantic coding in the right hemisphere (Kounios & Beeman 2014).

Latent learning refers to learning without immediate reinforcement, as compellingly demonstrated by Tolman’s maze experiments, in which rats that freely explored a maze managed to learn the spatial layout and later navigated it efficiently after a reward was introduced (Tolman 1948). This process underlies our ability to form cognitive maps and indicates that the brain can encode spatial relations independently of direct reinforcement, with the hippocampus playing a key role (O’Keefe & Nadel 1978). Such findings underscore the broader significance of spatial learning and map formation in guiding adaptive behavior.

One-shot learning—the ability to acquire a new concept from just a single exposure—has been demonstrated in behavioral tasks (Lake et al. 2011), with neuroimaging studies revealing that the hippocampus becomes selectively engaged during these rapid learning episodes (Lee et al. 2015). Rule-based and declarative learning involve the acquisition of knowledge through cognitive processes that support memory recall and abstract reasoning rather than relying primarily on temporal regularities. Finally, conceptual learning, such as forming abstract notions such as “justice,” and emotionally driven associative learning, wherein a single salient event such as a traumatic incident produces enduring changes, further expand the repertoire of nontemporal learning.

Together, these diverse forms of learning demonstrate that the brain acquires knowledge not solely through temporal regularities but also by detecting spatial, relational, and abstract patterns, ultimately constructing robust and flexible representations that guide adaptive behavior.

### 6.4. How Might Studies of Atypical Sensory Rearing Shed Light on Questions Related to the Development of Temporal Processing?

It is well-established that infants exploit various temporal regularities across several domains, such as vision (Leslie 1984, Aslin & Shea 1990, Fiser & Aslin 2002) and language (Saffran et al. 1996, Aslin et al. 1998, Pelucchi et al. 2009). Thus, from an early age, human infants, and the young of many species, are likely tuned to regularities in their environments, forming the building blocks of future perceptual and conceptual knowledge. In the visual domain specifically, while several studies reviewed above highlight the early use of temporal regularities, the rapid pace of visual development (Ayzenberg & Behrmann 2024) and operational challenges in studying newborns make it difficult to precisely determine what visual skills precede, and might causally influence, the development of other visual skills. A unique opportunity to examine such processes at the earliest stages of development arises through the study of rare groups of individuals with atypical development—those who were born blind and received sight-restoring surgeries later in childhood (Sinha 2013). Immediately and longitudinally following their sight-restoring surgeries, one can assess their performance on tasks probing simultaneity, contiguity, and contingency detection and explore how temporal cues bind and organize sensory inputs. Notably, such studies also provide important tests of developmental plasticity beyond traditional critical periods (Mitchell & Maurer 2022).

The general importance and resilience of temporal processing in the late-sighted have already been highlighted by past work. For instance, temporal contrast sensitivity is markedly more resilient to visual deprivation than spatial sensitivity (Ellemberg et al. 1999b, Ye et al. 2021), and motion facilitates intraobject integration in late-sighted individuals (Ostrovsky et al. 2009), with biological motion recovering robustly (Ben-Ami et al. 2022, Rajendran et al. 2020). Moreover, motion cues enhance facial expression recognition (Gilad-Gutnick et al. 2024) and support the perception of shapes from temporal information (Orlov et al. 2021). These studies suggest that temporal information may play a role in bootstrapping perceptual development postsurgery.

Future research could address several additional questions across the different timescales highlighted in this review. For instance, how do the late-sighted children’s temporal binding windows, measured in simultaneity and/or temporal order judgment tasks, compare with those of typically sighted controls? Do these windows narrow progressively, as observed in typical infancy (Lewkowicz & Flom 2014), and are there differences between unimodal and multimodal settings? Over longer temporal intervals, how well-developed is the perception of temporal causality in late-sighted children, and how robust is their ability to detect correlations and probabilistic relationships within and across senses? What is the relative timeline of the postoperative development of simultaneity, contiguity, and contingency detection, and how does it relate to that of other visual skills? Such examinations not only would provide new insights into the development of several time-based visual skills following treatment for congenital visual deprivation but might also constrain the hypothesis space about what visual skills may drive the development of other visual skills.

## 7. CONCLUSION

We suggest that our sensorium is organized, not chaotic, in large part because the brain excels at extracting and exploiting temporal regularities from the environment. Across visual, auditory, tactile, olfactory, and multisensory domains, temporal correlations serve as critical cues for binding stimuli into perceptually meaningful units. This process is fundamental to segmenting continuous sensory flux into distinct objects and events, thereby potentially resolving the challenge inherent in William James’s characterization of the neonatal “blooming, buzzing confusion.” Neural mechanisms, such as synaptic plasticity, oscillatory phase alignment, and predictive coding, underscore a biological commitment to temporal pattern recognition, highlighting it as a possible unifying principle that spans species and sensory modalities to support perception, learning, and action.

From an evolutionary perspective, it is unsurprising that organisms depend heavily on temporal regularities. The natural world is full of rhythms, from diurnal cycles to the periodic wingbeats of an approaching predator, all carrying critical predictive information. Natural selection has thus honed neural systems that detect these patterns, conferring significant survival advantages. Even in complex behaviors, such as language, music, and tool use, which are arguably hallmarks of human cognition, temporal pattern extraction remains indispensable.

Taken together, to navigate their worlds, organisms must first parse them, and leveraging temporal information constitutes a particularly powerful strategy for doing so. The ubiquitous presence of temporal regularities provides the brain with a system for predicting and grouping incoming signals, transforming the cacophony of raw sensory stimuli into a coherent, dynamic tapestry. If the story of cognitive science is, in part, a narrative of uncovering the brain’s hidden strategies, then the detection of temporal regularities stands out as one of its most elegant and general solutions to the puzzle of organizing the sensorium.

## References

[R1] Abbott LF, Blum KI. 1996. Functional significance of long-term potentiation for sequence learning and prediction. Cereb. Cortex 6(3):406–16 8670667 10.1093/cercor/6.3.406

[R2] Alais D, Blake R, Lee SH. 1998. Visual features that vary together over time group together over space. Nat. Neurosci. 1(2):160–64 10195133 10.1038/414

[R3] Alomar K, Aysel HI, Cai X. 2024. RNNs, CNNs and transformers in human action recognition: a survey and a hybrid model. Preprint, arXiv:2407.06162 [cs.CV]

[R4] Alzubaidi L, Zhang J, Humaidi AJ, Al-Dujaili A, Duan Y. 2021. Review of deep learning: concepts, CNN architectures, challenges, applications, future directions. J. Big Data 8:53 33816053 10.1186/s40537-021-00444-8 PMC8010506

[R5] Amadeo MB, Esposito D, Escelsior A, Campus C, Inuggi A, 2022. Time in schizophrenia: a link between psychopathology, psychophysics and technology. Transl. Psychiatry 12(1):331 35961974 10.1038/s41398-022-02101-x PMC9374791

[R6] Artieda J, Pastor MA, Lacruz F, Obeso JA. 1992. Temporal discrimination is abnormal in Parkinson’s disease. Brain 115(1):199–210 1559154 10.1093/brain/115.1.199

[R7] Aslin RN, Saffran JR, Newport EL. 1998. Computation of conditional probability statistics by 8-month-old infants. Psychol. Sci. 9(4):321–24

[R8] Aslin RN, Shea SL. 1990. Velocity thresholds in human infants: implications for the perception of motion. Dev. Psychol. 26(4):589–98

[R9] Ayzenberg V, Behrmann M. 2024. Development of visual object recognition. Nat. Rev. Psychol. 3(2):73–90

[R10] Babineau D, Longtin A, Lewis JE. 2006. Modeling the electric field of weakly electric fish. J. Exp. Biol. 209(18):3636–51 16943504 10.1242/jeb.02403

[R11] Baldwin DA, Kosie JE. 2021. How does the mind render streaming experience as events? Topics Cogn. Sci. 13(1):79–105 10.1111/tops.12502 32529736

[R12] Bangasser DA, Waxler DE, Santollo J, Shors TJ. 2006. Trace conditioning and the hippocampus: the importance of contiguity. J. Neurosci. 26(34):8702–6 16928858 10.1523/JNEUROSCI.1742-06.2006 PMC3289537

[R13] Barlow H. 1987. Cerebral cortex as model builder. In Matters of Intelligence: Conceptual Structures in Cognitive Neuroscience, ed. Vaina LM. Springer Netherlands

[R14] Barlow HB. 1989. Unsupervised learning. Neural Comput. 1(3):295–311

[R15] Barlow HB. 1994. What is the computational goal of the neocortex? In Large-Scale Neuronal Theories of the Brain, ed. Koch C, Davis JL. MIT Press

[R16] Batty C. 2014. Olfactory objects. In Perception and Its Modalities, ed. Stokes D, Matthen M, Biggs S. Oxford University Press

[R17] Baumann O, Vromen JM, Cheung A, McFadyen J, Ren Y, Guo CC. 2018. Neural correlates of temporal complexity and synchrony during audiovisual correspondence detection. eNeuro 5(1):ENEURO.0294-17.2018 10.1523/ENEURO.0294-17.2018 PMC5773885 29354682

[R18] Ben-Ami S, Gupta P, Yadav M, Shah P, Talwar G, 2022. Human (but not animal) motion can be recognized at first sight—after treatment for congenital blindness. Neuropsychologia 174:108307 35752267 10.1016/j.neuropsychologia.2022.108307

[R19] Bender MB. 1952. Disorders in Perception; with Particular Reference to the Phenomena of Extinction and Displacement. Charles C. Thomas

[R20] Bender MB, Feldman M, Sobin AJ. 1968. Palinopsia. Brain 91(2):321–38 5721933 10.1093/brain/91.2.321

[R21] Bender MB, Furlow LT. 1945. Phenomenon of visual extinction in homonymous fields and psychologic principles involved. Arch. Neurol. Psychiatry 53(1):29–33

[R22] Bender MB, Teuber HL. 1946. Phenomena of fluctuation, extinction and completion in visual perception. Arch. Neurol. Psychiatry 55(6):627–58 10.1001/archneurpsyc.1946.02300170075008 20990293

[R23] Bi GQ, Poo MM. 1998. Synaptic modifications in cultured hippocampal neurons: dependence on spike timing, synaptic strength, and postsynaptic cell type. J. Neurosci. 18(24):10464–72 9852584 10.1523/JNEUROSCI.18-24-10464.1998 PMC6793365

[R24] Bilger HT, Vertosick E, Vickers A, Kaczmarek K, Prum RO. 2021. Higher-order musical temporal structure in bird song. Front. Psychol. 12:629456 33868093 10.3389/fpsyg.2021.629456 PMC8044833

[R25] Binder M. 2015. Neural correlates of audiovisual temporal processing—comparison of temporal order and simultaneity judgments. Neuroscience 300:432–47 25982561 10.1016/j.neuroscience.2015.05.011

[R26] Bliss TV, Gardner-Medwin AR. 1973. Long-lasting potentiation of synaptic transmission in the dentate area of the unanaesthetized rabbit following stimulation of the perforant path. J. Physiol. 232(2):331–56 4727084 10.1113/jphysiol.1973.sp010273 PMC1350458

[R27] Bliss TV, Lømo T. 1973. Long-lasting potentiation of synaptic transmission in the dentate area of the anaesthetized rabbit following stimulation of the perforant path. J. Physiol. 232(2):331–56 4727084 10.1113/jphysiol.1973.sp010273 PMC1350458

[R28] Bloch G, Bar-Shai N, Cytter Y, Green R. 2017. Time is honey: circadian clocks of bees and flowers and how their interactions may influence ecological communities. Philos. Trans. R. Soc. B 372(1734):20160256 10.1098/rstb.2016.0256 PMC5647282 28993499

[R29] Bregman AS. 1994. Auditory Scene Analysis. The Perceptual Organization of Sound. MIT Press

[R30] Bruns P. 2019. The ventriloquist illusion as a tool to study multisensory processing: an update. Front. Integr. Neurosci. 13:51 31572136 10.3389/fnint.2019.00051 PMC6751356

[R31] Buehner MJ. 2005. Contiguity and covariation in human causal inference. Learn. Behav. 33(2):230–38 16075841 10.3758/bf03196065

[R32] Burnett S. 2011. Perceptual worlds and sensory ecology. Nat. Educ. Knowl. 3(10):75

[R33] Burr D, Silva O, Cicchini GM, Banks MS, Morrone MC. 2009. Temporal mechanisms of multimodal binding. Proc. R. Soc. B 276(1663):1761–69 10.1098/rspb.2008.1899 PMC2674495 19324779

[R34] Bushara KO, Grafman J, Hallett M. 2001. Neural correlates of auditory-visual stimulus onset asynchrony detection. J. Neurosci. 21(1):300–4 11150347 10.1523/JNEUROSCI.21-01-00300.2001 PMC6762435

[R35] Buzsáki G. 2002. Theta oscillations in the hippocampus. Neuron 33(3):325–40 11832222 10.1016/s0896-6273(02)00586-x

[R36] Cacchione T, Amici F. 2015. Cohesion as a principle for perceiving objecthood: Does it apply to animate agents? Swiss J. Psychol. 74(4):217–28

[R37] Cannon J, O’Brien AM, Bungert L, Sinha P. 2021. Prediction in autism spectrum disorder: a systematic review of empirical evidence. Autism Res. 14(4):604–30 33570249 10.1002/aur.2482 PMC8043993

[R38] Caporale N, Dan Y. 2008. Spike timing-dependent plasticity: a Hebbian learning rule. Annu. Rev. Neurosci. 31:25–46 18275283 10.1146/annurev.neuro.31.060407.125639

[R39] Carey S. 2009. The Origin of Concepts. Oxford University Press

[R40] Cheries EW, Mitroff SR, Wynn K, Scholl BJ. 2008. Cohesion as a constraint on object persistence in infancy. Dev. Sci. 11:427–32 18466376 10.1111/j.1467-7687.2008.00687.x PMC3665331

[R41] Ciullo V, Spalletta G, Caltagirone C, Jorge RE, Piras F. 2016. Explicit time deficit in schizophrenia: systematic review and meta-analysis indicate it is primary and not domain specific. Schizophr. Bull. 42(2):505–18 26253596 10.1093/schbul/sbv104 PMC4753592

[R42] Cowan N. 2010. The magical mystery four: How is working memory capacity limited, and why? Curr. Dir. Psychol. Sci. 19(1):51–57. 10.1177/0963721409359277 20445769 PMC2864034

[R43] Cox D, Meier P, Oertelt N, DiCarlo J. 2005. Breaking position invariant object recognition. Nat. Neurosci. 8:1145–47 16116453 10.1038/nn1519

[R44] Critchley M. 1951. Types of visual perseveration: “paliopsia” and “illusory visual spread”. Brain 74(3):267–99 14869536 10.1093/brain/74.3.267

[R45] Curtis CE, D’Esposito M. 2003. Persistent activity in the prefrontal cortex during working memory. Trends Cogn. Sci. 7(9):415–23 12963473 10.1016/s1364-6613(03)00197-9

[R46] Dan Y, Poo MM. 2004. Spike timing-dependent plasticity of neural circuits. Neuron 44(1):23–30 15450157 10.1016/j.neuron.2004.09.007

[R47] de Diego-Balaguer R, Rodríguez-Fornells A, Bachoud-Lévi AC. 2015. Prosodic cues enhance rule learning by changing speech segmentation mechanisms. Front. Psychol. 6:1478 26483731 10.3389/fpsyg.2015.01478 PMC4588126

[R48] Diamond SP, Bender MB. 1965. On auditory extinction and alloacusis. Trans. Am. Neurol. Assoc. 90:154–57 5857734

[R49] Dobson V, Teller DY. 1978. Visual acuity in human infants: a review and comparison of behavioral and electrophysiological studies. Vis. Res. 18(11):1469–83 364823 10.1016/0042-6989(78)90001-9

[R50] Dusenbery DB. 1992. Sensory Ecology: How Organisms Acquire and Respond to Information. W. H. Freeman & Company

[R51] Eichenbaum H. 2014. Time cells in the hippocampus: a new dimension for mapping memories. Nat. Rev. Neurosci. 15(11):732–44 25269553 10.1038/nrn3827 PMC4348090

[R52] Ellemberg D, Lewis TL, Liu CH, Maurer D. 1999a. Development of spatial and temporal vision during childhood. Vis. Res. 39(14):2325–33 10367054 10.1016/s0042-6989(98)00280-6

[R53] Ellemberg D, Lewis TL, Maurer D, Lui CH, Brent HP. 1999b. Spatial and temporal vision in patients treated for bilateral congenital cataracts. Vis. Res. 39(20):3480–89 10615511 10.1016/s0042-6989(99)00078-4

[R54] Engel AK, Fries P, Singer W. 2001. Dynamic predictions: oscillations and synchrony in top-down processing. Nat. Rev. Neurosci. 2(10):704–16 11584308 10.1038/35094565

[R55] Falter CM, Elliott MA, Bailey AJ. 2012. Enhanced visual temporal resolution in autism spectrum disorders. PLOS ONE 7(3):e32774 22470425 10.1371/journal.pone.0032774 PMC3309999

[R56] Fischer J, Whitney D. 2014. Serial dependence in visual perception. Nat. Neurosci. 17(5):738–43 24686785 10.1038/nn.3689 PMC4012025

[R57] Fischer S, Frommen JG. 2022. Predator detection. In Encyclopedia of Animal Cognition and Behavior, ed. Vonk J, Shackelford TK. Springer International Publishing

[R58] Fiser J, Aslin RN. 2002. Statistical learning of new visual feature combinations by infants. PNAS 99(24):15822–26 12429858 10.1073/pnas.232472899 PMC137800

[R59] Fitzwater ME, Reisman MN. 1952. Comparisons of forward, simultaneous, backward, and pseudo-conditioning. J. Exp. Psychol. 44(3):211–14 12990764 10.1037/h0060495

[R60] Földiák P. 1991. Learning invariance from transformation sequences. Neural Comput. 3(2):194–200 31167302 10.1162/neco.1991.3.2.194

[R61] Foucher JR, Lacambre M, Pham BT, Giersch A, Elliott MA. 2007. Low time resolution in schizophrenia: lengthened windows of simultaneity for visual, auditory and bimodal stimuli. Schizophr. Res. 97(1–3):118–27 17884350 10.1016/j.schres.2007.08.013

[R62] Freschl J, Melcher D, Carter A, Kaldy Z, Blaser E. 2021. Seeing a page in a flipbook: shorter visual temporal integration windows in 2-year-old toddlers with autism spectrum disorder. Autism Res. 14(5):946–58 33174396 10.1002/aur.2430 PMC8106666

[R63] Fries P. 2005. A mechanism for cognitive dynamics: neuronal communication through neuronal coherence. Trends Cogn. Sci. 9(10):474–80 16150631 10.1016/j.tics.2005.08.011

[R64] Friston K. 2010. The free-energy principle: a unified brain theory? Nat. Rev. Neurosci. 11(2):127–38 20068583 10.1038/nrn2787

[R65] Galef BG Jr., Giraldeau LA. 2001. Social influences on foraging in vertebrates: causal mechanisms and adaptive functions. Anim. Behav. 61(1):3–15 11170692 10.1006/anbe.2000.1557

[R66] Gerstner W, Kempter R, Van Hemmen JL, Wagner H. 1996. A neuronal learning rule for sub-millisecond temporal coding. Nature 383(6595):76–78 8779718 10.1038/383076a0

[R67] Giersch A. 2024. Time perception in neuropsychiatry. In Neural Bases of Timing and Time Perception, ed. Mioni G, Grondin S. Routledge

[R68] Giersch A, Lalanne L, Corves C, Seubert J, Shi Z, 2009. Extended visual simultaneity thresholds in patients with schizophrenia. Schizophr. Bull. 35(4):816–25 18359954 10.1093/schbul/sbn016 PMC2696372

[R69] Giersch A, Poncelet PE, Capa RL, Martin B, Duval CZ, 2015. Disruption of information processing in schizophrenia: the time perspective. Schizophr. Res. Cogn. 2(2):78–83 29114456 10.1016/j.scog.2015.04.002 PMC5609651

[R70] Gilad-Gutnick S, Kurian GS, Gupta P, Shah P, Tiwari K, 2024. Motion’s privilege in recognizing facial expressions following treatment for blindness. Curr. Biol. 34(17):4047–55 39116886 10.1016/j.cub.2024.07.046 PMC11457836

[R71] Glaze CM, Troyer TW. 2013. Development of temporal structure in zebra finch song. J. Neurophysiol. 109(4):1025–35 23175805 10.1152/jn.00578.2012 PMC3569136

[R72] Golesorkhi M, Gomez-Pilar J, Zilio F, Berberian N, Wolff A, 2021. The brain and its time: intrinsic neural timescales are key for input processing. Commun. Biol. 4(1):970 34400800 10.1038/s42003-021-02483-6 PMC8368044

[R73] Gusfield D. 1999. Algorithms on Strings, Trees and Sequences: Computer Science and Computational Biology. Cambridge University Press

[R74] Harman K, Humphrey G. 1999. Encoding ‘regular’ and ‘random’ sequences of views of novel three-dimensional objects. Perception 28:601–15 10664756 10.1068/p2924

[R75] Hebb DO. 1949. The Organization of Behavior. Wiley

[R76] Herzog MH, Drissi-Daoudi L, Doerig A. 2020. All in good time: long-lasting postdictive effects reveal discrete perception. Trends Cogn. Sci. 24(10):826–37 32893140 10.1016/j.tics.2020.07.001

[R77] Hespos SJ, vanMarle K. 2012. Physics for infants: characterizing the origins of knowledge about objects, substances, and number. WIREs Cogn. Sci. 3(1):19–27 10.1002/wcs.157 26302470

[R78] Hughes HC. 1999. Sensory Exotica: A World Beyond Human Experience. MIT Press

[R79] Hume D. 1888 (1739). A Treatise of Human Nature, ed. Selby-Bigge LA. Clarendon Press

[R80] Hut RA, Kronfeld-Schor N, van der Vinne V, De la Iglesia H. 2012. In search of a temporal niche: environmental factors. Prog. Brain Res. 199:281–304 22877672 10.1016/B978-0-444-59427-3.00017-4

[R81] Iglesias S, Mathys C, Brodersen KH, Kasper L, Piccirelli M, 2013. Hierarchical prediction errors in midbrain and basal forebrain during sensory learning. Neuron 80(2):519–30 24139048 10.1016/j.neuron.2013.09.009

[R82] Iversen IH. 1993. Techniques for establishing schedules with wheel running as reinforcement in rats. J. Exp. Anal. Behav. 60(1):219–38 8354968 10.1901/jeab.1993.60-219 PMC1322156

[R83] Ivry RB, Spencer RM. 2004. The neural representation of time. Curr. Opin. Neurobiol. 14(2):225–32 15082329 10.1016/j.conb.2004.03.013

[R84] Jacobs L, Feldman M, Diamond SP, Bender MB. 1973. Palinacousis: persistent or recurring auditory sensations. Cortex 9(3):275–87 4777741 10.1016/s0010-9452(73)80005-x

[R85] Jacquey L, Fagard J, Esseily R, O’Regan JK. 2020. Detection of sensorimotor contingencies in infants before the age of 1 year: a comprehensive review. Dev. Psychol. 56:1233–51 32463268 10.1037/dev0000916

[R86] James W. 1890. The Principles of Psychology. Henry Holt

[R87] Jenkins HM, Ward WC. 1965. Judgment of contingency between responses and outcomes. Psychol. Monogr. Gen. Appl. 79(1):1–17 10.1037/h0093874 14300511

[R88] Jesse A, Johnson EK. 2016. Audiovisual alignment of co-speech gestures to speech supports word learning in 2-year-olds. J. Exp. Child Psychol. 145:1–10 26765249 10.1016/j.jecp.2015.12.002

[R89] Johnston A, Nishida SY. 2001. Time perception: brain time or event time? Curr. Biol. 11(11):R427–30 11516664 10.1016/s0960-9822(01)00252-4

[R90] Kandil FI, Fahle M. 2001. Purely temporal figure–ground segregation. Eur. J. Neurosci. 13(10):2004–8 11403694 10.1046/j.0953-816x.2001.01573.x

[R91] Kellman PJ, Spelke ES. 1983. Perception of partly occluded objects in infancy. Cogn. Psychol. 15(4):483–524 6641127 10.1016/0010-0285(83)90017-8

[R92] Kimchi R, Behrmann M, Olson CR. 2003. Perceptual Organization in Vision: Behavioral and Neural Perspectives. Taylor & Francis

[R93] Kimura T, Kadota H, Kuroda T, Funai TD, Iwata M, 2019. Neural correlates of tactile simultaneity judgement: a functional magnetic resonance imaging study. Sci. Rep. 9(1):19481 31862896 10.1038/s41598-019-54323-7 PMC6925270

[R94] Kinsbourne M, Warrington EK. 1962. A disorder of simultaneous form perception. Brain 85(3):461–86 14032918 10.1093/brain/85.3.461

[R95] Kirkham NZ, Slemmer JA, Johnson SP. 2002. Visual statistical learning in infancy: evidence of a domain general learning mechanism. Cognition 83:B35–42 11869728 10.1016/s0010-0277(02)00004-5

[R96] Köhler W. 1925. The Mentality of Apes. Harcourt, Brace & Co

[R97] Kounios J, Beeman M. 2014. The cognitive neuroscience of insight. Annu. Rev. Psychol. 65:71–93 24405359 10.1146/annurev-psych-010213-115154

[R98] Krizhevsky A, Sutskever I, Hinton GE. 2017. ImageNet classification with deep convolutional neural networks. Commun. ACM 60(6):84–90

[R99] Lake B, Salakhutdinov R, Gross J, Tenenbaum J. 2011. One shot learning of simple visual concepts. In Proceedings of the 33rd Annual Meeting of the Cognitive Science Society, ed. Carlson L. Cognitive Science Society.

[R100] Lashley KS. 1951. The problem of serial order in behavior. In Cerebral Mechanisms in Behavior, ed Jeffress LA. Bobbs-Merrill

[R101] Lawson RP, Mathys C, Rees G. 2017. Adults with autism overestimate the volatility of the sensory environment. Nat. Neurosci. 20(9):1293–99 28758996 10.1038/nn.4615 PMC5578436

[R102] Lee SH, Blake R. 1999. Visual form created solely from temporal structure. Science 284:1165–68 10325226 10.1126/science.284.5417.1165

[R103] Lee SW, O’Doherty JP, Shimojo S. 2015. Neural computations mediating one-shot learning in the human brain. PLOS Biol. 13(4):e1002137 25919291 10.1371/journal.pbio.1002137 PMC4412411

[R104] Leslie AM. 1984. Spatiotemporal continuity and the perception of causality in infants. Perception 13(3):287–305 6514514 10.1068/p130287

[R105] Levy WB, Steward O. 1983. Temporal contiguity requirements for long-term associative potentiation/depression in the hippocampus. Neuroscience 8(4):791–97 6306504 10.1016/0306-4522(83)90010-6

[R106] Lewkowicz DJ. 2014. Early experience and multisensory perceptual narrowing. Dev. Psychobiol. 56(2):292–315 24435505 10.1002/dev.21197 PMC3953347

[R107] Lewkowicz DJ, Flom R. 2014. The audiovisual temporal binding window narrows in early childhood. Child Dev. 85(2):685–94 23888869 10.1111/cdev.12142 PMC3954953

[R108] Liu T. 2007. Learning sequence of views of three-dimensional objects: the effect of temporal coherence on object memory. Perception 36:1320–33 18196699 10.1068/p5778

[R109] Locke J. 1996 (1689). An Essay Concerning Human Understanding, ed. Winkler KP. Hackett Publishing Company

[R110] Love SA, Petrini K, Pernet CR, Latinus M, Pollick FE. 2018. Overlapping but divergent neural correlates underpinning audiovisual synchrony and temporal order judgments. Front. Hum. Neurosci. 12:274 30018545 10.3389/fnhum.2018.00274 PMC6037859

[R111] Lupyan G, Ward EJ. 2013. Language can boost otherwise unseen objects into visual awareness. PNAS 110(35):14196–201 23940323 10.1073/pnas.1303312110 PMC3761589

[R112] MacDonald CJ, Lepage KQ, Eden UT, Eichenbaum H. 2011. Hippocampal “time cells” bridge the gap in memory for discontiguous events. Neuron 71(4):737–49 21867888 10.1016/j.neuron.2011.07.012 PMC3163062

[R113] Manttari J, Broomé S, Folkesson J, Kjellstrom H. 2020. Interpreting video features: a comparison of 3D convolutional networks and convolutional LSTM networks. In Computer Vision – ACCV 2020, 15th Asian Conference on Computer Vision, Kyoto, Japan, November 30 – December 4, 2020, Revised Selected Papers, Part V, ed. Ishikawa H, Liu C-L, Pajdla T, Shi J. Springer

[R114] Markram H, Lübke J, Frotscher M, Sakmann B. 1997. Regulation of synaptic efficacy by coincidence of postsynaptic APs and EPSPs. Science 275(5297):213–15 8985014 10.1126/science.275.5297.213

[R115] Mau W, Sullivan DW, Kinsky NR, Hasselmo ME, Howard MW, Eichenbaum H. 2018. The same hippocampal CA1 population simultaneously codes temporal information over multiple timescales. Curr. Biol. 28:1499–508 29706516 10.1016/j.cub.2018.03.051 PMC5964012

[R116] McEchron MD, Bouwmeester H, Tseng W, Weiss C, Disterhoft JF. 1998. Hippocampectomy disrupts auditory trace fear conditioning and contextual fear conditioning in the rat. Hippocampus 8(6):638–46 9882021 10.1002/(SICI)1098-1063(1998)8:6<638::AID-HIPO6>3.0.CO;2-Q

[R117] Meredith MA, Stein BE. 1986. Visual, auditory, and somatosensory convergence on cells in superior colliculus results in multisensory integration. J. Neurophysiol. 56(3):640–62 3537225 10.1152/jn.1986.56.3.640

[R118] Michotte A. 1963. The Perception of Causality. Basic Books

[R119] Mitchell DE, Maurer D. 2022. Critical periods in vision revisited. Annu. Rev. Vis. Sci. 8:291–321 35385674 10.1146/annurev-vision-090721-110411

[R120] Miyashita Y. 1988. Neuronal correlate of visual associative long-term memory in the primate temporal cortex. Nature 335:817–20 3185711 10.1038/335817a0

[R121] Moss CF, Surlykke A. 2001. Auditory scene analysis by echolocation in bats. J. Acoust. Soc. Am. 110(4):2207–26 11681397 10.1121/1.1398051

[R122] Mota-Rojas D, Bienboire-Frosini C, Marcet-Rius M, Domínguez-Oliva A, Mora-Medina P, 2022. Mother-young bond in non-human mammals: neonatal communication pathways and neurobiological basis. Front. Psychol. 13:1064444 36524176 10.3389/fpsyg.2022.1064444 PMC9744768

[R123] Nakatani M, Kobayashi Y, Ohno K, Uesaka M, Mogami S, 2021. Temporal coherency of mechanical stimuli modulates tactile form perception. Sci. Rep. 11(1):11737 34083558 10.1038/s41598-021-90661-1 PMC8175693

[R124] O’Doherty JP, Dayan P, Friston K, Critchley H, Dolan RJ. 2004. Temporal difference models and reward-related learning in the human brain. Neuron 38(2):329–37 10.1016/s0896-6273(03)00169-7 12718865

[R125] O’Keefe J, Nadel L. 1978. The Hippocampus as a Cognitive Map. Clarendon Press

[R126] Orhan AE, Lake BM. 2024. Learning high-level visual representations from a child’s perspective without strong inductive biases. Nat. Mach. Intell. 6(3):271–83

[R127] Orlov T, Raveh M, McKyton A, Ben-Zion I, Zohary E. 2021. Learning to perceive shape from temporal integration following late emergence from blindness. Curr. Biol. 31(14):3162–67 34043950 10.1016/j.cub.2021.04.059

[R128] Ostrovsky Y, Meyers E, Ganesh S, Mathur U, Sinha P. 2009. Visual parsing after recovery from blindness. Psychol. Sci. 20(12):1484–91 19891751 10.1111/j.1467-9280.2009.02471.x

[R129] Parise CV, Ernst MO. 2016. Correlation detection as a general mechanism for multisensory integration. Nat. Comm. 7:11543 10.1038/ncomms11543 PMC4897755 27265526

[R130] Parise CV, Spence C, Ernst MO. 2012. When correlation implies causation in multisensory integration. Curr. Biol. 22(1):46–49 22177899 10.1016/j.cub.2011.11.039

[R131] Parker KL, Lamichhane D, Caetano MS, Narayanan NS. 2013. Executive dysfunction in Parkinson’s disease and timing deficits. Front. Integr. Neurosci 7:75 24198770 10.3389/fnint.2013.00075 PMC3813949

[R132] Pascucci D, Tanrikulu ÖD, Ozkirli A, Houborg C, Ceylan G, 2023. Serial dependence in visual perception: a review. J. Vis. 23(1):9 10.1167/jov.23.1.9 PMC9871508 36648418

[R133] Paterson M, Dančík V. 1994. Longest common subsequences. In Proceedings of the 19th International Symposium on Mathematical Foundations of Computer Science, ed. Prívara I, Rovan B, Ruzicka P. Springer Berlin Heidelberg

[R134] Pavlov IP. 1927. Conditioned Reflexes: An Investigation of the Physiological Activity of the Cerebral Cortex. Oxford University Press 10.5214/ans.0972-7531.1017309 PMC4116985 25205891

[R135] Pedroso SS, Barber I, Svensson O, Fonseca PJ, Amorim MCP. 2013. Courtship sounds advertise species identity and male quality in sympatric *Pomatoschistus* spp. gobies. PLOS ONE 8(6):e64620 23755129 10.1371/journal.pone.0064620 PMC3674009

[R136] Pelucchi B, Hay JF, Saffran JR. 2009. Statistical learning in a natural language by 8-month-old infants. Child Dev. 80(3):674–85 19489896 10.1111/j.1467-8624.2009.01290.x PMC3883431

[R137] Pezze MA, Marshall HJ, Cassaday HJ. 2016. Potentiation rather than distraction in a trace fear conditioning procedure. Behav. Process. 128:41–46 10.1016/j.beproc.2016.04.003 PMC4906245 27060226

[R138] Piaget J. 1952 (1935). The Origins of Intelligence in Children. Norton

[R139] Ptacek R, Weissenberger S, Braaten E, Klicperova-Baker M, Goetz M, 2019. Clinical implications of the perception of time in attention deficit hyperactivity disorder (ADHD): a review. Med. Sci. Monit. 25:3918–24 31129679 10.12659/MSM.914225 PMC6556068

[R140] Rajendran SS, Bottari D, Shareef I, Pitchaimuthu K, Sourav S, 2020. Biological action identification does not require early visual input for development. eNeuro 7(5):ENEURO.0534-19.2020 10.1523/ENEURO.0534-19.2020 PMC7598910 33060179

[R141] Rao RP, Ballard DH. 1999. Predictive coding in the visual cortex: a functional interpretation of some extra-classical receptive-field effects. Nat. Neurosci. 2(1):79–87 10195184 10.1038/4580

[R142] Rescorla RA. 1967. Pavlovian conditioning and its proper control procedures. Psychol. Rev. 74(1):71–80 5341445 10.1037/h0024109

[R143] Rescorla RA. 2008. Evaluating conditioning of related and unrelated stimuli using a compound test. Learn. Behav. 36(2):67–74 18543707 10.3758/lb.36.2.67

[R144] Rescorla R, Wagner A. 1972. A theory of Pavlovian conditioning: variations in the effectiveness of reinforcement and nonreinforcement. In Classical Conditioning II: Current Research And Theory, ed. Black AH, Prokasy WF. Appleton-Century-Crofts

[R145] Rosenberg RD, Carey S. 2009. Infants’ representation of material entities. In The Origins of Object Knowledge, ed. Hood BM, Santos LR. Oxford University Press

[R146] Rostami Z, Salari M, Mahdavi S, Etemadifar M. 2024. Abnormal multisensory temporal discrimination in Parkinson’s disease. Brain Res. 1834:148901 38561085 10.1016/j.brainres.2024.148901

[R147] Roth MJ, Lindner A, Hesse K, Wildgruber D, Wong HY, Buehner MJ. 2023. Impaired perception of temporal contiguity between action and effect is associated with disorders of agency in schizophrenia. PNAS 120(21):e2214327120 37186822 10.1073/pnas.2214327120 PMC10214164

[R148] Saffran JR, Aslin RN, Newport EL. 1996. Statistical learning by 8-month-old infants. Science 274(5294):1926–28 8943209 10.1126/science.274.5294.1926

[R149] Schultz W. 1998. Predictive reward signal of dopamine neurons. J. Neurophysiol. 80(1):1–27 9658025 10.1152/jn.1998.80.1.1

[R150] Sekuler R, Watamaniuk SN, Blake R. 2002. Motion perception. In Steven’s Handbook of Experimental Psychology, Vol. 1, ed. Yantis S. John Wiley & Sons

[R151] Shamma SA, Elhilali M, Micheyl C. 2011. Temporal coherence and attention in auditory scene analysis. Trends Neurosci. 34(3):114–23 21196054 10.1016/j.tins.2010.11.002 PMC3073558

[R152] Shanks DR, Pearson SM, Dickinson A. 1989. Temporal contiguity and the judgment of causality by human subjects. Q. J. Exp. Psychol. 41B(2):139–59

[R153] Singer W. 1999. Neuronal synchrony: a versatile code for the definition of relations? Neuron 24(1):49–65 10677026 10.1016/s0896-6273(00)80821-1

[R154] Singer W, Gray CM. 1995. Visual feature integration and the temporal correlation hypothesis. Annu. Rev. Neurosci. 18:555–86 7605074 10.1146/annurev.ne.18.030195.003011

[R155] Sinha P. 2013. Once blind and now they see. Sci. Am. 309:48–55 10.1038/scientificamerican0713-48 23821864

[R156] Sinha P, Balas BJ, Ostrovsky Y. 2009. Visual object discovery. In Object Categorization: Computer and Human Vision Perspectives, ed. Dickinson S, Tarr M. Cambridge University Press

[R157] Sinha P, Kjelgaard MM, Gandhi TK, Tsourides K, Cardinaux AL, 2014a. Autism as a disorder of prediction. PNAS 111(42):15220–25 25288765 10.1073/pnas.1416797111 PMC4210351

[R158] Sinha P, Poggio T. 1996. Role of learning in three-dimensional form perception. Nature 384(6608):460–63 8945472 10.1038/384460a0

[R159] Sinha P, Wulff J, Held R. 2014b. Establishing cross-modal mappings: empirical and computational investigations. In Sensory Integration and the Unity of Consciousness, ed. Bennett D, Hill C. MIT Press

[R160] Skinner BF. 1938. The Behavior of Organisms: An Experimental Analysis. Appleton-Century

[R161] Slater A, Mattock A, Brown E. 1990. Size constancy at birth: newborn infants’ responses to retinal and real size. J. Exp. Child Psychol. 49:314–22 2332727 10.1016/0022-0965(90)90061-c

[R162] Smith J. 1989. Senses & Sensibilities. John Wiley & Sons

[R163] Solomon PR, Vander Schaaf ER, Thompson RF, Weisz DJ. 1986. Hippocampus and trace conditioning of the rabbit’s classically conditioned nictitating membrane response. Behav. Neurosci. 100(5):729–44 3778636 10.1037//0735-7044.100.5.729

[R164] Song S, Miller KD, Abbott LF. 2000. Competitive Hebbian learning through spike-timing-dependent synaptic plasticity. Nat. Neurosci. 3(9):919–26 10966623 10.1038/78829

[R165] Spelke ES. 1994. Initial knowledge: six suggestions. Cognition 50:431–45 8039373 10.1016/0010-0277(94)90039-6

[R166] Spelke ES, Kinzler KD. 2007. Core knowledge. Dev. Sci. 10:89–96 17181705 10.1111/j.1467-7687.2007.00569.x

[R167] Spooner A, Kellogg WN. 1947. The backward conditioning curve. Am. J. Psychol. 60(3):321–34 20254693

[R168] Stephan KE, Mathys C. 2014. Computational approaches to psychiatry. Curr. Opin. Neurobiol. 25:85–92 24709605 10.1016/j.conb.2013.12.007

[R169] Sternberg DA, McClelland JL. 2012. Two mechanisms of human contingency learning. Psychol. Sci. 23(1):59–68 22198929 10.1177/0956797611429577

[R170] Sutton RS. 1988. Learning to predict by the methods of temporal differences. Mach. Learn. 3:9–44

[R171] Sutton RS, Barto AG. 1998. Reinforcement Learning: An Introduction. MIT Press

[R172] Szyszka P, Emonet T, Edwards TL. 2023. Extracting spatial information from temporal odor patterns: insights from insects. Curr. Opin. Insect Sci. 59:101082 37419251 10.1016/j.cois.2023.101082 PMC10878403

[R173] Taxidis J, Pnevmatikakis EA, Dorian CC, Mylavarapu AL, Arora JS, 2020. Differential emergence and stability of sensory and temporal representations in context-specific hippocampal sequences. Neuron 108:984–98 32949502 10.1016/j.neuron.2020.08.028 PMC7736335

[R174] Tolman EC. 1948. Cognitive maps in rats and men. Psychol. Rev. 55(4):189–208 18870876 10.1037/h0061626

[R175] Tosini G, Bertolucci C, Foà A. 2001. The circadian system of reptiles: a multioscillatory and multiphotoreceptive system. Physiol. Behav. 72(4):461–71 11282129 10.1016/s0031-9384(00)00423-6

[R176] Turk-Browne NB, Scholl BJ, Chun MM, Johnson MK. 2009. Neural evidence of statistical learning: efficient detection of visual regularities without awareness. J. Cogn. Neurosci. 21(10):1934–45 18823241 10.1162/jocn.2009.21131 PMC2773825

[R177] Usher M, Donnelly N. 1998. Visual synchrony affects binding and segmentation in perception. Nature 394(6689):179–82 9671300 10.1038/28166

[R178] Vogelsang L, Gilad-Gutnick S, Ehrenberg E, Yonas A, Diamond S, 2018. Potential downside of high initial visual acuity. PNAS 115(44):11333–38 30322940 10.1073/pnas.1800901115 PMC6217435

[R179] Vogelsang L, Vogelsang M, Pipa G, Diamond S, Sinha P. 2024. Butterfly effects in perceptual development: a review of the ‘adaptive initial degradation’ hypothesis. Dev. Rev. 71:101117 40838216 10.1016/j.dr.2024.101117 PMC12363605

[R180] Vogelsang M, Vogelsang L, Diamond S, Sinha P. 2023. Prenatal auditory experience and its sequelae. Dev. Sci. 26(1):e13278 35583318 10.1111/desc.13278 PMC11164537

[R181] Vogelsang M, Vogelsang L, Gupta P, Gandhi TK, Shah P, 2024. Impact of early visual experience on later usage of color cues. Science 384(6698):907–12 38781366 10.1126/science.adk9587 PMC12226138

[R182] von der Emde G. 1999. Active electrolocation of objects in weakly electric fish. J. Exp. Biol. 202(10):1205–15 10210662 10.1242/jeb.202.10.1205

[R183] von Helmholtz H. 1867. Handbuch der Physiologischen Optik, Vol. 9. Voss

[R184] Vong WK, Wang W, Orhan AE, Lake BM. 2024. Grounded language acquisition through the eyes and ears of a single child. Science 383(6682):504–11 38300999 10.1126/science.adi1374

[R185] Wallis G, Backus BT, Langer M, Huebner G, Bülthoff H. 2009. Learning illumination- and orientation-invariant representations of objects through temporal association. J. Vis. 9(7):6 19761321 10.1167/9.7.6

[R186] Wallis G, Bülthoff HH. 2001. Effects of temporal association on recognition memory. PNAS 98(8):4800–4 11287633 10.1073/pnas.071028598 PMC31914

[R187] Watabe-Uchida M, Eshel N, Uchida N. 2017. Neural circuitry of reward prediction error. Annu. Rev. Neurosci. 40:373–94 28441114 10.1146/annurev-neuro-072116-031109 PMC6721851

[R188] Watson JS. 1966. The development and generalization of “contingency awareness” in early infancy: some hypotheses. Merrill Palmer Q. Behav. Dev. 12:123–35

[R189] Watson JS. 1984. Bases of causal inference in infancy: time, space and sensory relations. In Advances in Infancy Research, Vol. 3, ed. Lipsitt LP, Rovee-Collier C. Ablex

[R190] Wertheimer M. 1923. Untersuchungen zur Lehre von der Gestalt. II. Psychol. Forsch. 4:301–50

[R191] Wu Y, Chen K, Xing C, Huang M, Zhao K, Zhou W. 2024. Human olfactory perception embeds fine temporal resolution within a single sniff. Nat. Hum. Behav. 8(11):2168–78 39402256 10.1038/s41562-024-01984-8

[R192] Wundt W. 1913. Grundriss der Psychologie. Kröner

[R193] Ye J, Gupta P, Shah P, Tiwari K, Gandhi T, 2021. Resilience of temporal processing to early and extended visual deprivation. Vis. Res. 186:80–86 34062374 10.1016/j.visres.2021.05.004 PMC8858639

[R194] Zaadnoordijk L, Besold TR, Cusack R. 2022. Lessons from infant learning for unsupervised machine learning. Nat. Mach. Intell. 4(6):510–20

[R195] Zacks JM, Swallow KM. 2007. Event segmentation. Curr. Dir. Psychol. Sci. 16(2):80–84 22468032 10.1111/j.1467-8721.2007.00480.x PMC3314399

[R196] Zhou HY, Cai XL, Weigl M, Bang P, Cheung EF, Chan RC. 2018. Multisensory temporal binding window in autism spectrum disorders and schizophrenia spectrum disorders: a systematic review and meta-analysis. Neurosci. Biobehav. Rev. 86:66–76 29317216 10.1016/j.neubiorev.2017.12.013

